# Hydroxyl-Rich Hydrophilic Endocytosis-Promoting Peptide with No Positive Charge

**DOI:** 10.1021/jacs.2c07420

**Published:** 2022-10-27

**Authors:** Siwen Wang, Zhonghan Li, Desiree Aispuro, Nathan Guevara, Juno Van Valkenburgh, Boxi Chen, Xiaoyun Zhou, Matthew N. McCarroll, Fei Ji, Xu Cong, Priyanka Sarkar, Rohit Chaudhuri, Zhili Guo, Nicole P. Perkins, Shiqun Shao, Jason K. Sello, Kai Chen, Min Xue

**Affiliations:** † Department of Chemistry, University of California, Riverside, California 92521, United States; ‡ Department of Radiology, Keck School of Medicine, University of Southern California, Los Angeles, California 90033, United States; § Department of Pharmaceutical Chemistry, University of California, San Francisco, California 94143, United States; ∥ College of Chemical and Biological Engineering, Zhejiang University, Hangzhou, Zhejiang 310027, P. R. China

## Abstract

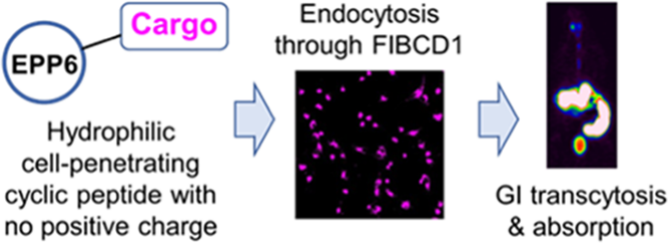

Delivering cargo molecules across the plasma membrane is critical for biomedical research, and the need to develop molecularly well-defined tags that enable cargo transportation is ever-increasing. We report here a hydrophilic endocytosis-promoting peptide (EPP6) rich in hydroxyl groups with no positive charge. EPP6 can transport a wide array of small-molecule cargos into a diverse panel of animal cells. Mechanistic studies revealed that it entered the cells through a caveolin- and dynamin-dependent endocytosis pathway, mediated by the surface receptor fibrinogen C domain-containing protein 1. After endocytosis, EPP6 trafficked through early and late endosomes within 30 min. Over time, EPP6 partitioned among cytosol, lysosomes, and some long-lived compartments. It also demonstrated prominent transcytosis abilities in both *in vitro* and *in vivo* models. Our study proves that positive charge is not an indispensable feature for hydrophilic cell-penetrating peptides and provides a new category of molecularly well-defined delivery tags for biomedical applications.

## Introduction

Transporting cargo molecules into the cell is a critical and everlasting need in many biomedical studies.^1−3^ As a natural route to bring molecules into the cell, the endocytosis process has been a primary focus in the field, especially when hydrophilic cargo molecules are involved.^1,3,4^ A broad spectrum of endocytosis-promoting modalities has been discovered and developed. For instance, nanoparticles, such as liposomes and micelles,^5,6^ can serve as delivery vehicles and bring native-state cargo molecules across the cell membrane.^3^ Some nanoparticle delivery platforms have already demonstrated clinical success.^3,6,7^ On the other hand, smaller endocytosis-promoting moieties provide a more molecularly well-defined approach. These delivery tags allow for a more straightforward manufacturing process and a clearer path for medicinal chemistry optimizations than nanoparticle-based platforms. Representative examples of these groups include folate,^8^ transferrin,^9^ miniature proteins,^10^ and, notably, cell-penetrating peptides (CPPs).^11−13^

Over the past few decades, a very diverse panel of CPPs has been established. Early examples such as the transactivator of transcription (TAT) peptide^14^ and RGD sequence^15^ have proven capable of delivering various cargo molecules, and they continue to be widely employed to date.^11,12^ More recently, advanced sequences, such as penetratin, iRGD, CPP12, cTAT, and miniature proteins, have demonstrated superior delivery efficacy.^11−13,16,17^ Currently, improving the cell-penetrating ability, intracellular targeting, and biocompatibility of these CPPs remains a very active and attractive research field.

Despite the prominent sequence variations, these hydrophilic CPPs share a common feature—they are positively charged. This positive charge promotes the initial interaction with the extracellular matrix and the cell membrane, which is a prerequisite for endocytosis.^11−13,16^ Consequently, the current dogma regards this positive charge as an indispensable component of hydrophilic CPPs. However, considering that cells can efficiently take up heavily negatively charged nanoparticles, one may question the necessity of relying on the positive charge to induce CPP endocytosis. Herein, we present an endocytosis-promoting peptide (EPP) that has no positive charge. This cyclic peptide is rich in hydroxyl groups, and we show that it can bring various cargo molecules into a diverse collection of animal cells.

## Results

### Hydroxyl-Rich Cyclic Peptides Bring Cargo Molecules into Cells

**Figure 1:**
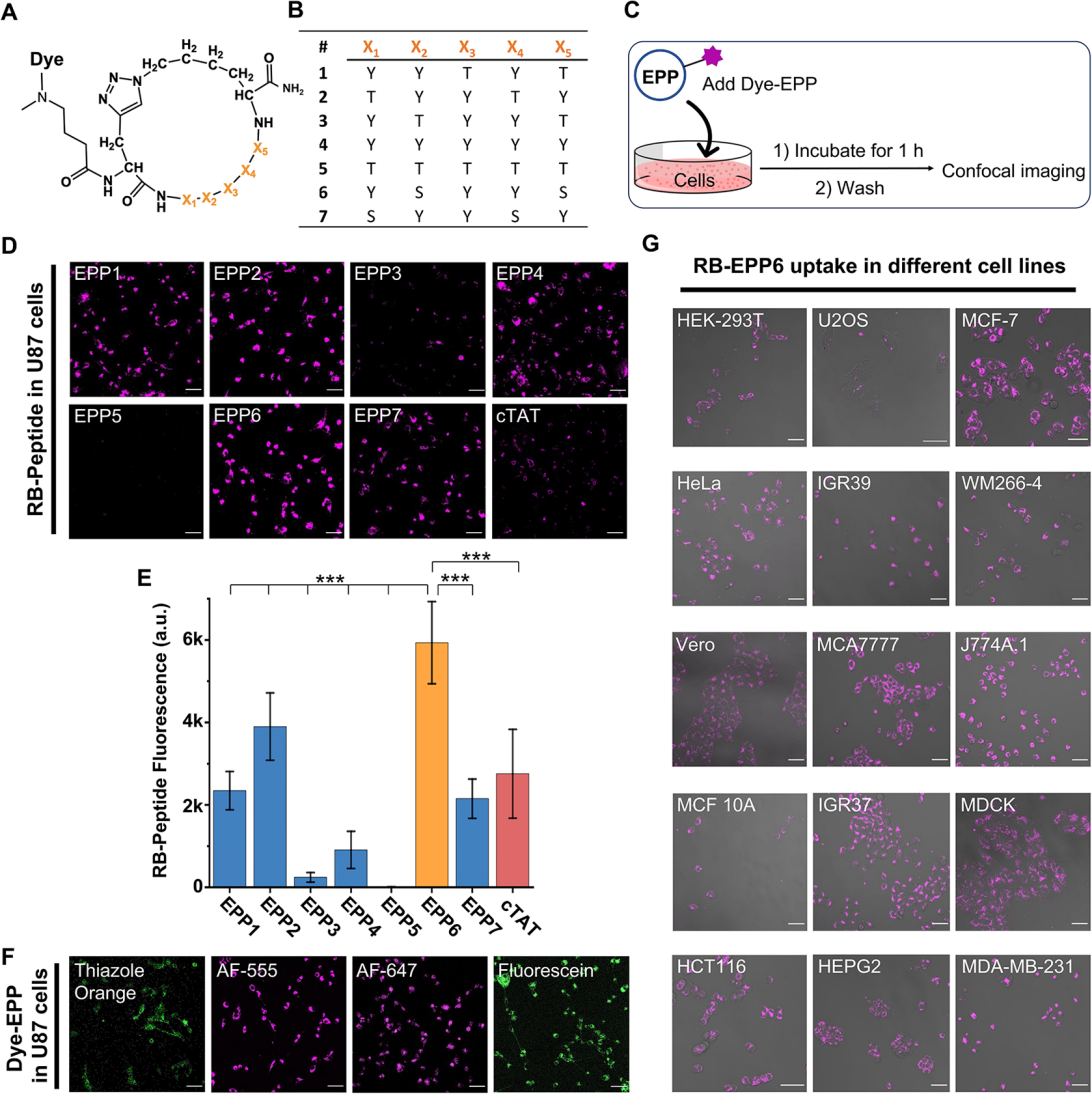
Hydroxyl-rich cyclic peptides facilitate the transportation of cargo molecules into cells. The scale bars are 50 μm. (A) Generic structure of the EPPs. (B) Sequences of EPP1-7. (C) General method for assessing dye-EPP uptake. (D) Confocal images showing that EPPs were able to bring the RB group into U87 cells. (E) Single-cell intracellular fluorescence intensities quantified from the confocal images. The error bars denote standard deviations. 50 representative cells were extracted in each image. RB-cTAT was used for comparison. ***p < 0.001 by Mann–Whitney test. (F) Confocal images showing that EPP6 was able to bring different cargo molecules into the cells. (G) Confocal images showing that RB-EPP6 could enter a wide panel of cell lines.

Inspired by the endocytosis-promoting properties of glycans,^18^ we prepared seven hydroxyl-rich cyclic peptides using tyrosine, threonine, and serine as the building blocks. These peptides contain five modular amino acids and a triazole ring cyclized through a CuAAC reaction (Figure 1A,B). The exposed N terminal was designed for cargo conjugation. In our first example, we linked these peptides to a rhodamine B (RB) tag (Figure S1). We chose the U87 cell line as the model system and used confocal imaging to evaluate the uptake of RB-EPPs (Figure 1C). As shown in Figure 1D, these peptides had different abilities to enter the cells, with EPP6 demonstrating the highest efficiency. This observation was consistent with flow cytometry results (Figure S2). Compared with the benchmark, cyclic TAT (cTAT) peptide,^17^ EPP6 led to ∼2X fluorescence intensity in the cells. More interestingly, we found that RB-EPP6 uptake was concentration-dependent across a wide range without affecting cell viability (Figures S3 and S4). In our subsequent studies, we focused on EPP6 as the model compound.

Considering that the size of EPP6 and the RB tag was comparable and RB was known to interact with the cell membrane,^19^ we must confirm that EPP6, rather than the RB tag, was the critical component for the uptake. Therefore, we conjugated EPP6 to different fluorescent dye moieties and performed similar uptake experiments. Our results proved that EPP6 could bring all these cargo molecules into U87 cells (Figure 1F). It is worth pointing out that AF555 and AF647 are negatively charged dyes and exhibit negligible interaction with the cell membrane.^19^ Therefore, our results confirmed that it was the EPP6 moiety that enabled the uptake.

We then sought to test if the observed RB-EPP6 uptake was limited to U87 cells. As shown in Figure 1G, EPP6 was able to enter a wide array of human cancer and noncancer cells as well as non-human cells. Nevertheless, the resulting intracellular fluorescence intensities varied significantly across different cell lines, and we also found a few cell lines that did not take up EPP6 efficiently (Figure S5), which prompted us to investigate the mechanism of cell entry.

### EPP6 Enters the Cells through Caveolin- and Dynamin-Dependent Endocytosis

Unlike the well-studied positively charged CPPs (TAT, penetrating, poly-R, CPP12, *etc.*),^11,16,20^ our EPPs do not have charged residues. On the other hand, the hydroxyl groups may form intramolecular hydrogen bonds that render the EPPs hydrophobic, which could confer membrane permeability.^21^ To this end, we performed octanol partitioning experiments to assess the hydrophobicity of RB-EPPs (Figure S6A). Interestingly, most of the RB-EPPs were not strongly hydrophobic (Figure S6B). The best-performing candidate, RB-EPP6, was slightly hydrophilic, while the worst one, RB-EPP3, was the most hydrophobic. These results proved that hydrophobicity was not the driving force in EPP uptake.

**Figure 2:**
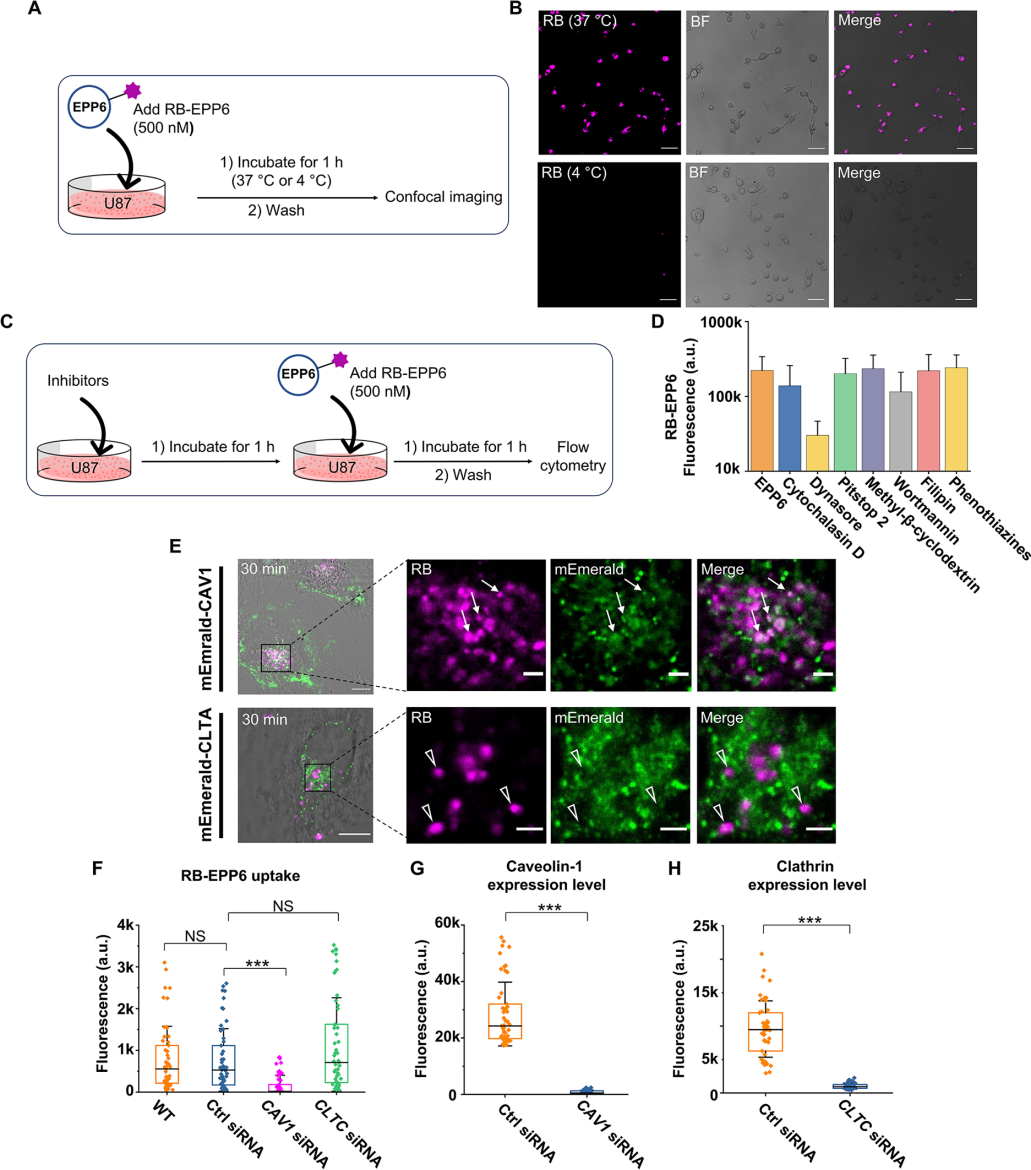
EPP6 entered the cell through a dynamin- and caveolin-dependent process. (A,B) RB-EPP6 uptake at different temperatures (B) showing that low-temperature incubation led to no RB-EPP6 uptake in U87 cells. Scale bars: 50 μm. (C,D) Flow cytometry results showing the effects of inhibitors on the uptake of RB-EPP6 in U87 cells. Error bars: standard deviations. (E) Confocal images showing the overlap (arrows) between mEmerald-CAV1 and RB-EPP6 and the lack of colocalization (triangles) between mEmerald-CLTA and RB-EPP6. Scale bars: 10 μm (original) and 2 μm (zoom-in). (F) Caveolin-1 (*CAV-1*) knockdown caused decreased RB-EPP6 uptake in U87 cells, while clathrin (*CLTC*) knockdown did not affect the uptake. The wildtype sample was not treated with siRNA, and the control siRNA sample was treated with a scrambled siRNA that did not target the human genome. 53 single-cell data points were extracted from confocal images. (G,H) Immunofluorescence results comparing the caveolin-1and clathrinexpression levels between the ctrl siRNA sample (51 data points) and the *CAV1* (51 data points) and *CLTC* (45 data points) siRNA-treated U87 cells. ***Mann–Whitney *p* < 0.001; NS, not significant, *p* > 0.05; boxes, middle two quartiles; horizontal lines, median levels; whiskers, standard deviations.

To test whether EPPs entered the cells through passive diffusion or active transportation, we evaluated how temperature affected the uptake (Figure 2A). As shown in Figure 2B, the low-temperature treatment led to no fluorescence signal in the cells, suggesting an energy-dependent uptake process.^16,20^ To validate our findings, we performed parallel artificial membrane permeability assay (PAMPA).^22^ We found that EPPs could not pass through the artificial membrane (Table S1), which indicated that EPPs entered the cells through active transportation mechanisms.

Considering the molecule size of the EPPs and that different dye conjugates were all able to enter the cells, we reasoned that the transportation was unlikely to involve transmembrane transport proteins or ion channels. Therefore, an endocytosis pathway was more plausible. To identify critical components in EPP transportation, we studied the effects of endocytosis inhibitors on RB-EPP6 uptake (Figure 2C). As shown in Figure 2D (and Figure S7), dynamin inhibition (Dynasore) almost completely suppressed RB-EPP6 uptake (86% inhibition).^23^ Similarly, inhibition of actin polymerization (cytochalasin D)^24^ and PI3K signaling (wortmannin)^25^ also significantly impeded RB-EPP6 uptake by 38 and 48%, respectively. In contrast, inhibitors against clathrin-mediated endocytosis (Pitstop 2^26^ and phenothiazine^27^) did not strongly affect the uptake (9% and no inhibition, respectively), which pointed to a clathrin-independent process. Based on the current understanding of endocytosis pathways, our results hinted at a caveolin-mediated endocytosis pathway.^28,29^

To further delineate the roles of caveolin and clathrin, we sought to fluorescently label caveolin and clathrin and evaluate their spatial relationship with EPP6. To this end, we transfected U87 cells with mEmerald-CAV1^30^ and mEmerald-CLTA plasmids.^31^ These plasmids have been widely used to monitor the location and assembly of caveolin and clathrin. Using the transfected cells, we observed that RB-EPP6 signals significantly overlapped with the mEmerald-caveolin fluorescence, while no overlap existed between RB-EPP6 and mEmerald-CLTA (Figure 2E). Moreover, we found that caveolin-1 (*CAV1*) knockdown by siRNA resulted in a significant decrease of RB-EPP6 uptake, while clathrin (*CLTC*) knockdown had no effects (Figures 2F–H and S8–S10). These results further validated our hypothesis that EPP6 entered the cells through a caveolin-dependent pathway.

Interestingly, we further found that cholesterol extraction (methyl-β-cyclodextrin) and cholesterol binding (filipin) did not inhibit RB-EPP6 uptake (Figure 2D).^28^ These results were unusual because caveolin-mediated endocytosis mechanisms typically involve cholesterol and lipid rafts.^28^ Another common pathway of caveolin-dependent endocytosis relies on heparin sulfate proteoglycans (HSPGs), which is implicated in the uptake of some well-established CPPs and many other macromolecules.^32^ However, high concentrations of heparin sulfate did not affect RB-EPP6 uptake (Figure S11), which proved that HSPG was not involved. Taken together, our results suggested that EPP6 took an uncommon dynamin- and caveolin-dependent endocytosis pathway, perhaps through a receptor-mediated endocytosis process.

### FIBCD1 is a Surface Receptor for EPP6 Recognition

**Figure 3:**
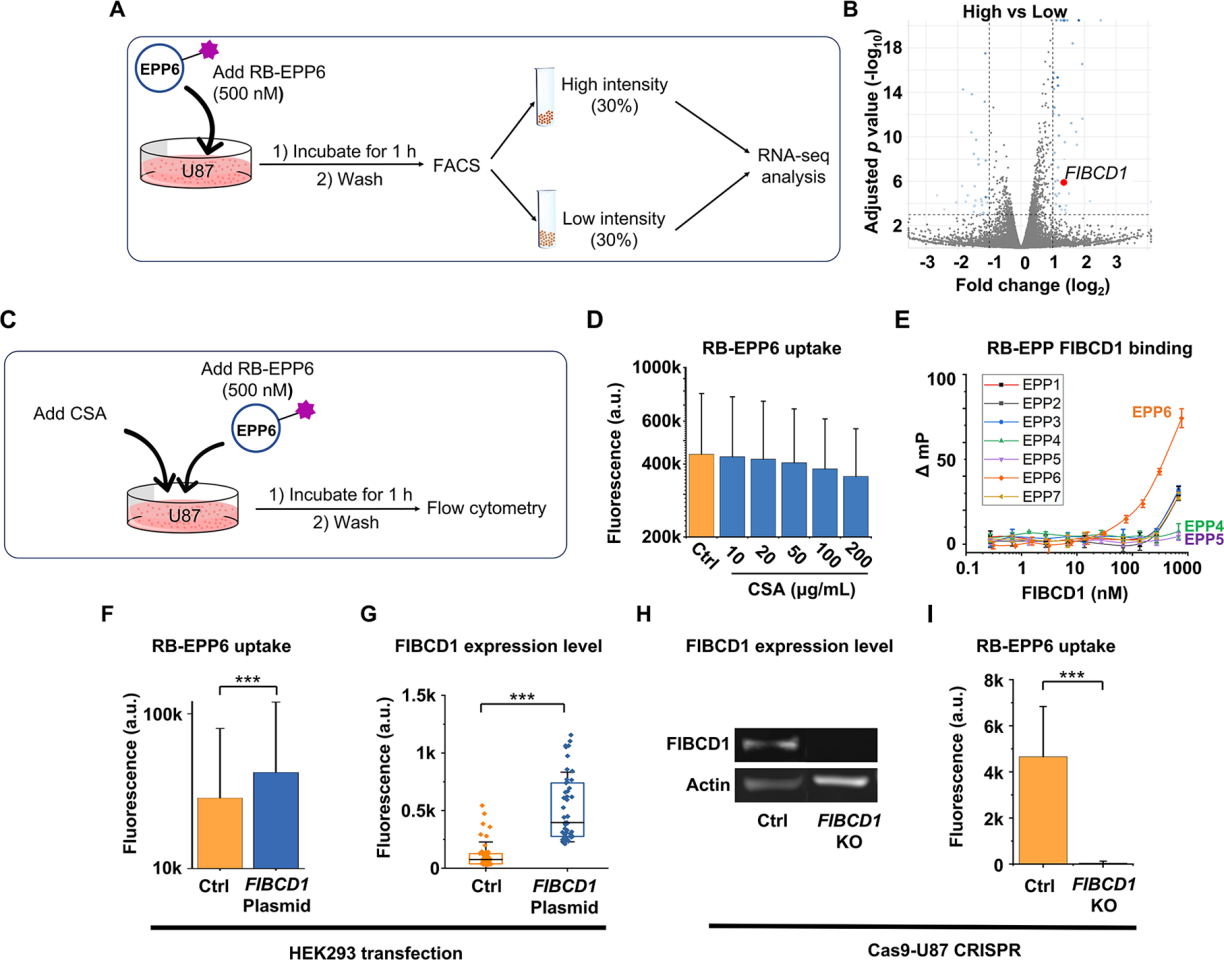
FIBCD1 is a surface receptor for EPP6 recognition. (A) Schematic illustration of the FACS and RNA-seq experimental procedure. (B) RNA-seq results identifying FIBCD1 as the potential receptor for EPP6. The horizontal dot line represents the *p* = 0.001 cutoff. The vertical dot lines represent 2-fold changes in expression levels. (C) Schematic illustration of the experiment for comparing RB-EPP6 uptake in the presence of CSA. (D) Flow cytometry results showing the effects of CSA treatment on the uptake of RB-EPP6 in U87 cells. (E) RB-EPP FIBCD1 fluorescence polarization assay results showing that RB-EPP6 has the highest binding affinity toward recombinant FIBCD1. (F) Flow cytometry results showing that FIBCD1 overexpression led to increased RB-EPP6 uptake in HEK293 cells. (G) Immunofluorescence results showing the increased FIBCD1 expression levels after plasmid transfection in HEK293 cells. 50 representative single-cell data points were extracted from each sample. (H) Western blot results showing that the *FIBCD1* gene was successfully knocked out by CRISPR in Cas9-U87 cells. (I) Confocal imaging results showing that *FIBCD1*-KO by CRISPR obliterated RB-EPP6 uptake in Cas9-U87 cells. 86 representative single-cell data points were extracted from images for each sample. Mann–Whitney tests were used to evaluate the statistical significance. ***p < 0.001.

To better understand the EPP6 endocytosis mechanism, we resorted to identifying the genes involved in the uptake. We incubated U87 cells with RB-EPP6 and used a fluorescence-activated cell sorter to isolate the top 30% and the bottom 30% populations based on the RB fluorescence. RNAs from these two populations were extracted for transcriptome analysis by RNA-seq (Figure 3A). We prepared three biological repeats for each condition and obtained, on average, 30 million reads per sample. The raw data were processed using fastp,^33^ STAR,^34^ and featureCounts^35^ to generate annotated gene lists, and the differentially expressed genes between the samples were identified by DESeq2.^36^ Here, we aimed to find upregulated genes in the high-uptake samples that could be receptors for EPP6 uptake.

Based on the criteria of *p* < 0.001 and > 2-fold changes, we found 38 genes that were upregulated (Figure 3B and Table S2). A closer examination revealed several cell surface protein candidates, which could be potential receptors for EPP6. One gene, fibrinogen C domain-containing protein 1 (FIBCD1), stood out, and we hypothesized that it was the receptor responsible for EPP6 uptake.

FIBCD1 is a type II transmembrane receptor known to induce endocytosis.^37^ Its identified ligands include mono- and oligosaccharides, such as chondroitin sulfate A (CSA), acetylmannosamine, chitin, β-1,3-glucan, and galactomannan.^38,39^ Notably, significant similarities exist between these ligands and the hydroxyl-rich EPPs as they all present abundant hydroxyl groups. Indeed, results from a competitive uptake experiment (Figure 3C)^38^ showed that CSA was able to inhibit RB-EPP6 uptake in a concentration-dependent manner (Figures 3D and S12A), without affecting cell viability (Figure S12B), supporting the hypothesis that FIBCD1 was responsible for EPP6 uptake. In addition, cell lines (Caco2 and Jurkat, Figure S5)^40^ that did not exhibit EPP6 uptake also lacked FIBCD1 expression.^41^

To validate FIBCD1’s role in EPP uptake, we set to assess the binding affinity between the EPPs and FIBCD1. We overexpressed full-length FIBCD1 in CHO cells, extracted and purified the protein, and performed fluorescence polarization assays to obtain the binding affinities. As shown in Figure 3E, EPP6 exhibited the strongest binding to FIBCD1, with a *K*_d_ around 200 nM. The other EPPs showed weaker binding, consistent with their inferior uptake results (Figure 1E).

Moreover, we found that FIBCD1 overexpression by plasmid transfection significantly increased RB-EPP6 uptake in HEK293 cells (Figures 3F,G and S13). To further validate our hypothesis, we leveraged the CRISPR technology and performed *FIBCD1*-KO in a cas9-expressing U87 stable cell line (Figures 3H and S14). We found that RB-EPP6 uptake was obliterated completely in these *FIBCD1*-KO cells (Figures 3I and S15). To rule out potential influences of stable cas9 expression, we also performed similar experiments in wildtype U87 cells treated with FIBCD1-CRISPR/Cas9KO-GFP plasmids and observed consistent results (Figure S16). These findings strongly proved that FIBCD1 was a receptor for EPP6 uptake.

### Intracellular Fate of EPP6

**Figure 4:**
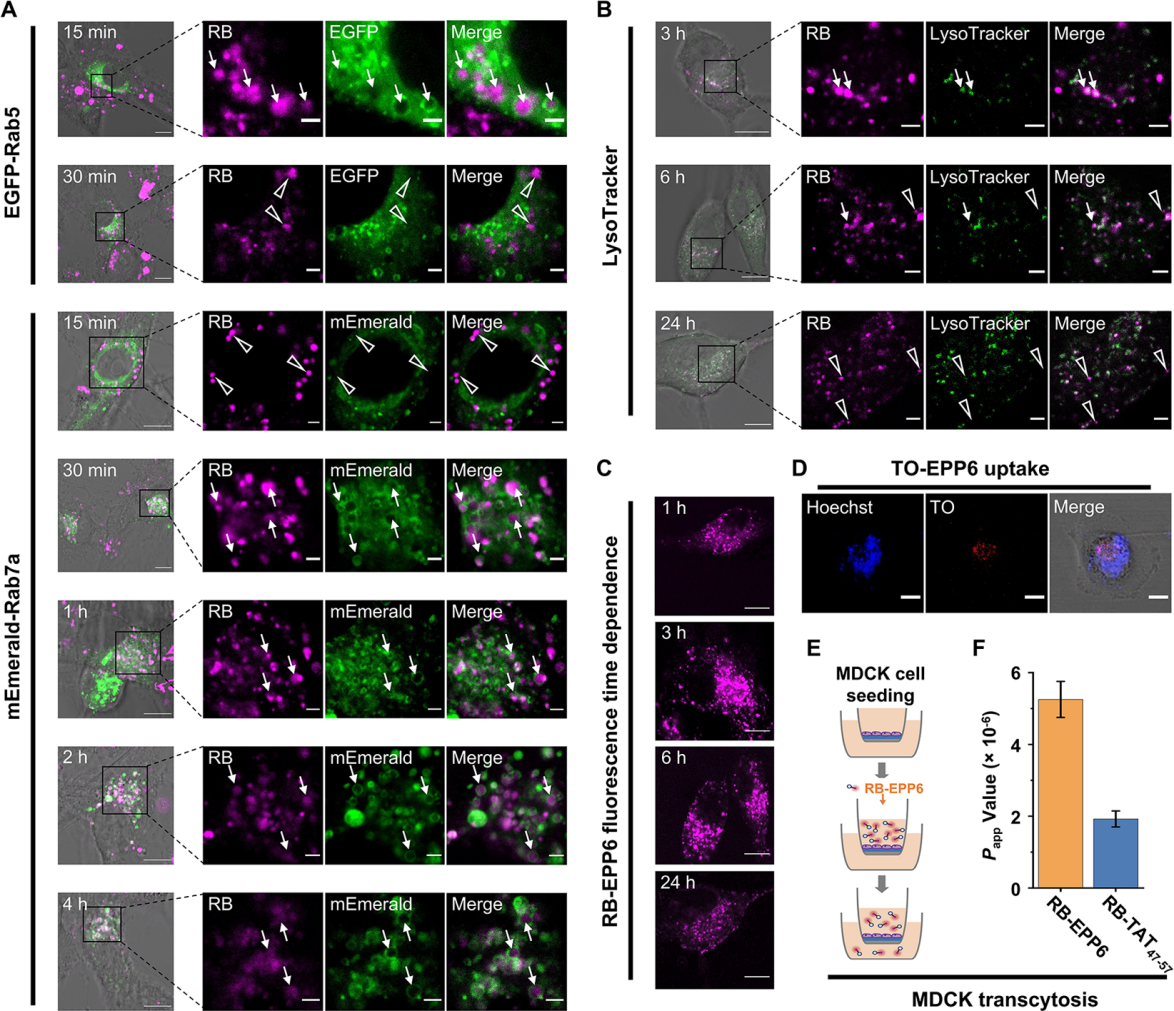
Intracellular fate of EPP6. The scale bars show 10 μm (original) and 2 μm (zoom-in). Arrows show colocalized signals, and triangles show un-colocalized signals. (A) Representative confocal images showing the trafficking of RB-EPP6 from Rab5+ to Rab7a+ vesicles within 30 min. (B) Representative confocal images showing time-dependent overlaps between lysosomes and RB-EPP6. (C) Representative confocal images showing RB-EPP6 signals in U87 cells in a time-dependent manner. (D) Representative confocal images showing thiazole orange-labeled EPP6 (TO-EPP6) accumulated in the nuclear region of U87 cells. (E) Schematic illustration of the MDCK monolayer transcytosis assay. (F) RB-EPP6 and RB-TAT Papp values obtained from the MDCK transcytosis assay. The error bars show standard deviations of four biological repeats.

We sought to investigate the fate of EPP6 after endocytosis. Typically, cargo molecules go through the early endosome—late endosome—lysosome pathway after endocytosis, and we hypothesized that EPP6 would adopt a similar pathway. To label the endosomes, we transfected U87 cells with EGFP-Rab5 (early endosome marker) and mEmerald-Rab7a (late endosome marker). We incubated the transfected cells with RB-EPP6 and used confocal microscopy to analyze the colocalization of RB-EPP6 and the fluorescent markers. As shown in Figure 4A, RB-EPP6 appeared in distinct Rab5-coated vesicles after 15 min, indicating that RB-EPP6 traveled into early endosomes quickly after endocytosis. Meanwhile, no apparent colocalization was observed between Rab7a-labeled vesicles and RB-EPP6. At 30 min, RB-EPP6 disappeared from the Rab5-labeled vesicles and appeared in Rab7a-labeled vesicles. This transition suggested that RB-EPP6 trafficked into late endosomes within 30 min. Interestingly, we found that a significant amount of RB-EPP6 remained inside Rab7a+ vesicles even after 4 h.

To further investigate the intracellular trafficking of RB-EPP6 after endocytosis, we used LysoTracker to label the lysosomes and assessed the signal colocalization. We found that some RB-EPP6 signals appeared in lysosomes after 3 h (Figure 4B). Nevertheless, many RB-EPP6-containing vesicles were not in lysosomes even after 6 h, which was consistent with our results that the RB-EPP6 signal remained in Rab7a+ vesicles. Interestingly, some punctate signals remained even after 24 h (Figure 4C), suggesting that some RB-EPP6 trafficked in long-lived vesicles or compartments. We also validated that RB-EPP6 did not move into the mitochondria or the endoplasmic reticulum (Figure S17).

In addition to the punctate signals, we also observed a significantly smeared signal that suggested cytosolic distribution of EPP6. To validate this finding, we prepared thiazole orange (TO-EPP6) and ethidium bromide (EB-EPP6) conjugates and investigated their intracellular fates. TO and EB have relatively low fluorescence in solution but exhibit significantly enhanced fluorescence signals upon binding to DNA and RNA. As shown in Figures 4D and S18, prominent TO-EPP6 signals appeared in the nucleus (indicated by Hoechst staining). 3D reconstruction of high-resolution z-stack confocal images also confirmed the colocalization (Supporting Information movies). Similarly, we observed EB-EPP6 signal in the nucleus in a concentration-dependent manner (Figure S19). Because endocytosed EPP6 must exit the endosome to access the nuclear region, our results proved the cytosolic partitioning of EPP6.

It was also obvious that the total intracellular RB-EPP6 fluorescence intensities decreased over time (Figure 4C). Considering that FIBCD1 was indicated in immune recognition,^39^ we hypothesized that some of the cargo molecules might exit the cells through exocytosis, which might further enable transcytosis. To test this hypothesis, we prepared tight MDCK cell monolayers in a trans-well apparatus and assessed the apical-to-basal transcytosis rate of RB-EPP6 (Figure 4E).^42^ After 3 h of incubation, we found a Papp value of 5.25 × 10^–6^ for RB-EPP6, which was more than twice of that of RB-TAT_47–57_ (Figure 4F).

### *In Vivo* Fate of EPP6

**Figure 5:**
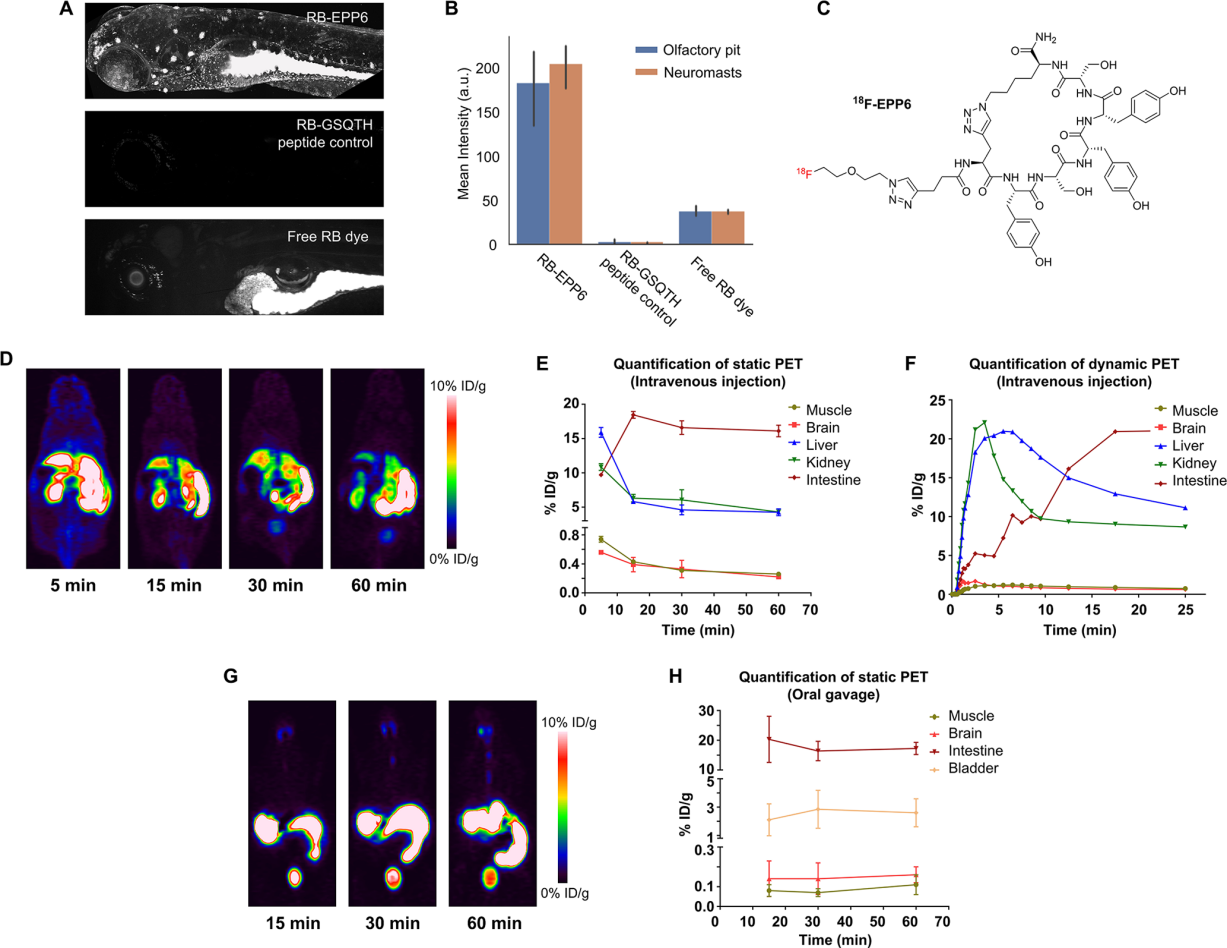
*In vivo* biodistribution of EPP6. (A) Representative fluorescence microscopy images of zebrafish larvae (6 days after fertilization) treated with RB-EPP6, EB-GSQTH (peptide control), and free RB dye. RB-EPP6 preferentially accumulated in olfactory pits and neuromasts, with slight accumulation in the vasculature in zebrafish larvae (6 days after fertilization). (B) Comparison of fluorescence intensity at the olfactory pits and neuromasts under different treatment conditions. (C) Structure of ^18^F-EPP6. (D) Representative whole-body PET imaging of nude mice at 5, 15, 30, and 60 min after intravenous injection of ^18^F-EPP6. (E) Quantification of static PET in major tissues/organs of nude mice after intravenous injection of ^18^F-EPP6. (F) Quantification of dynamic PET in major tissues/organs of nude mice after intravenous injection of ^18^F-EPP6. (G) Representative whole-body PET imaging of nude mice at 15, 30, and 60 min after oral gavage of ^18^F-EPP6. (H) Quantification of static PET in the major tissues/organs of nude mice after oral gavage of ^18^F-EPP6.

To study the properties of EPP6 *in vivo*, we tested the biodistribution of EPP6 on a zebrafish larvae model. We incubated the larvae (6 days post fertilization, 6dpf) with RB-EPP6 for 1 h and evaluated the resulting RB fluorescence. Because human FIBCD1 expression was associated with ciliated cells, we expected to see labeling of similar tissues in zebrafish. As shown in Figure 5A, RB-EPP6 preferentially accumulated in the olfactory pit and neuromasts rich in ciliated epithelial cells compared with the free rhodamine dye and the RB-GSQTH peptide control (Figure 5B). We also saw a prominent accumulation of RB-EPP6 in the gut and a slight accumulation in the vasculature (Figure 5A).

We then moved on to assess the biodistribution of EPP6 in mouse models. We administered ^18^F-labeled EPP6 (^18^F-EPP6, Figures 5C and S20–S24) into nude mice *via* intravenous injection (i.v., tail vein) or oral gavage. We then employed positron emission tomography (PET) to monitor the biodistribution of ^18^F-EPP6 *in vivo*. As shown in Figure 5D, EPP6 accumulated rapidly in the liver, kidneys, and intestine (5 min after i.v. injection). After 60 min, the intestine became the dominant accumulation site (Figure 5D) at ∼15 % ID/g (Figure 5E). Dynamic PET results (Figure 5F) dovetailed the results and showed that EPP6 accumulated in the liver and kidneys in less than 5 min, followed by a rapid decline. On the other hand, accumulation in the intestine occurred over 15 min, and the signal was maintained afterward. Similarly, oral administration of EPP6 led to a prominent accumulation in the intestine (Figure 5G,H). Interestingly, renal clearance was also evident in this case. This result indicates that EPP6 was quickly absorbed through the intestine and successfully entered the circulation, which underscored the transcytosis activities of EPP6.

## Discussion

Molecularly well-defined delivery tags can bring cargo molecules into cells through endocytosis, and they have profound research and therapeutic applications. Because their sizes are often comparable to, if not smaller than, the cargo, the delivery efficiency is cargo-dependent.^8,10,12,13,43^ Consequently, there are no universally effective delivery tags for all cargos, and there remains a pressing need to expand the arsenal of delivery tags. The EPP6 reported here represents a new paradigm. It is hydrophilic, uncharged, and able to transport a wide array of small-molecule cargos into a diverse panel of animal cells.

The lack of charge is the crucial feature differentiating EPP6 from other conventional CPPs. All existing hydrophilic CPPs contain positive charges, which are deemed necessary for the initial steps of endocytosis.^12,16^ Nevertheless, the premise of this notion is that positively charged CPPs interact with negatively charged membrane components such as lipids and HSPGs, which is only a fraction of the mechanisms that can trigger endocytosis. Therefore, it is natural to hypothesize that peptide sequences without positive charges may also trigger endocytosis, most likely through unique receptor-mediated pathways. Indeed, our EPP6 results support this hypothesis and prove that positive charge is not an indispensable part of hydrophilic CPPs.

Our study found that EPP6 entered cells through FIBCD1-mediated endocytosis in a caveolin- and dynamin-dependent manner. FIBCD1 is implicated in pathogen recognition, where it binds to saccharides.^39^ It is known to induce endocytosis, but the detailed mechanism remains elusive.^37^ Our results indicate that FIBCD1-mediated endocytosis required actin, dynamin, and caveolin, painting a clearer picture of the FIBCD1 endocytic process. On the other hand, our findings also underscore the complexity of endocytosis pathways, especially the caveolin-dependent ones.^28^ Existing studies emphasize the critical role of lipid rafts in caveolin-dependent endocytosis as the membrane enrichment of caveolin relies on these cholesterol-rich regions.^28,44^ However, we found that cholesterol-binding agents (filipin and methyl-cyclodextrin) did not affect RB-EPP6 uptake, proving that caveolin-dependent endocytosis can also take a lipid raft-independent route. In addition, our results echo the emerging opinion that the specificity of endocytosis inhibitors should be carefully evaluated.^45^

Our results have delineated the intracellular fate of EPP6. We believe that the endocytosed EPP6 first goes through Rab5+ and Rab7a+ endosomes and then partitions among the cytosol, lysosome, as well as some long-lived nonlysosomal compartments. Some of the endocytosed EPP6 is also transported out of the cell through transcytosis. Nevertheless, a few connecting pieces remain missing from the picture. For instance, the identity of the long-lived vesicles is unknown, and the mechanism of endosomal escape is unclear. More work needs to be done to elucidate the dictating factors that govern the fate of endocytosed EPP6, as well as strategies to control and adjust the fates as needed.

The presented EPP6 has immediate therapeutic implications. Because the expression of FIBCD1 varies significantly across different tissues and organs, it is also possible to leverage this difference and tailor EPP6 delivery for specific targets. For instance, the high expression levels of FIBCD1 and caveolin in the digestive tract may allow efficient gastrointestinal drug delivery and absorption. Indeed, a strong accumulation of EPP6 in the intestine was observed in both zebrafish and mice models (Figure 5A,D,G), and EPP6 was able to pass through the intestine lining and enter the circulation.^46^ More intriguingly, the Papp value of RB-EPP6 (5.3 × 10^–6^) is higher than the cutoff threshold of central nervous system availability (3.5 × 10^–6^),^42^ which hints at the potential of crossing the blood–brain barrier. Although we only observed very minute signals in the brain (Figure 5E,H), it remains hopeful that further iterations based on EPP6 may achieve better brain-targeting ability. Finally, our discovery of EPP6 encourages us to identify more tags targeting different endocytic receptors, which will further diversify the arsenal of delivery tags for research and therapeutic applications.

## Conclusions

We presented a hydroxyl-rich cyclic peptide (EPP6) that can promote endocytosis and deliver cargo molecules into a wide range of cells. EPP6 is distinct from conventional CPPs as it contains no positive charge. We identified FIBCD1 as the surface receptor for EPP6 and found that the endocytosis is dynamin-dependent, caveolin-dependent, and clathrin-independent. After endocytosis, EPP6 partitions among cytosol, lysosome, and some long-lived compartments. It also exhibits prominent transcytosis. We envision that EPP6, as well as its iterations, can enable and potentiate a broad range of drug-delivery methods.

## Supporting Information Available

Experimental details, materials and methods, HPLC and mass spectrometry characterization of synthesized peptides, additional confocal microscopy images and flow cytometry results, octanol partitioning experiment results, uncropped western blot raw image, PAMPA assay results, and upregulated gene list from RNA-seq analysis (PDF)
TO-EPP6 Hoechst 3D Z Stack render (AVI)
TO-EPP6 Hoechst Z Stack slice (AVI)

## Supplementary Material

ja2c07420_si_001.pdf

ja2c07420_si_002.avi

ja2c07420_si_003.avi
