# Supplementary material for: Hydroxyl-Rich Hydrophilic Endocytosis-Promoting Peptide with No Positive Charge
Source: J Am Chem Soc. 2022 Oct 27;144(44):20288–97. doi: 10.1021/jacs.2c07420 (PMC9650711; doi:10.1021/jacs.2c07420)
Supplement: Supplementary file 1 — ja2c07420_si_001.pdf [file ja2c07420_si_001.pdf]

# Hydroxyl-Rich Hydrophilic Endocytosis-Promoting Peptide with No Positive Charge

Siwen Wang[†], Zhonghan Li[†], Desiree Aispuro[†], Nathan Guevara[†], Juno Van Valkenburgh[‡], Boxi Chen[†], Xiaoyun Zhou[‡], Matthew N. McCarroll[$], Fei Ji[†], Xu Cong[†], Priyanka Sarkar[†], Rohit Chaudhuri[†], Zhili Guo[†], Nicole P. Perkins[†], Shiqun Shao[†, #], Jason K. Sello[$], Kai Chen[‡, *], and Min Xue[†, *]

[†]Department of Chemistry, University of California, Riverside, Riverside, California 92521, United States

[‡]Department of Radiology, Keck School of Medicine, University of Southern California, Los Angeles, California 90033, United States

[$]Department of Pharmaceutical Chemistry, University of California, San Francisco, San Francisco, California 94143, United States

[#]College of Chemical and Biological Engineering, Zhejiang University, Hangzhou, Zhejiang 310027, P.R. China

* Corresponding authors.
Email: chenkai@med.usc.edu (K.C.), minxue@ucr.edu (M.X.)

Supporting Information

## MATERIALS AND REAGENTS

TentaGel S-$NH_2$ resin (loading capacity 0.28 mmol/g) was purchased from Rapp Polymere GmbH and Rink amide MBHA resin (loading capacity 0.678 mmol/g) from Aapptec (Louisville, KY). All the Fmoc-protected amino acids were purchased from Anaspec (Fremont, CA) except Fmoc-L-propargylglycine (Pra) and Fmoc-Lys($N_3$)-OH (Az4), which were purchased from Chempep (Wellington, FL) and Chem-Impex (Wood Dale, IL), respectively. The coupling reagent 2-(1*H*-benzotriazol-1-yl)-1,1,3,3-tetramethyluronium hexafluorophosphate (HBTU, 99.6%) was obtained from Chem-Impex (Wood Dale, IL). Diisopropylethylamine (DIEA, 99.5%) was purchased from ACROS (Germany). Phenyl isothiocyanate (PhNCS) and triisopropylsilane (TIPS) were obtained from TCI (Portland, OR). Piperidine was purchased from Alfa Aesar (Ward Hill, MA). 5(6)-carboxyfluorescein (Fluo) was obtained from ACROS (Pittsburg, PA). Rhodamine B (RB), cuprous iodide (CuI), ethidium bromide (EB), α-cyano-4-hydroxycinnamic acid (CHCA), and anti-FIBCD1 antibody (AV50135) were obtained from Sigma-Aldrich (St. Louis, MO). *N*,*N'*-dimethylformamide (DMF), dichloromethane (DCM), control siRNA, *CAV1*-siRNA (Silencer® Select, s2446), and *CLTC*-siRNA (s475) were purchased from Thermo Fisher Scientific (Waltham, MA). Heparin sodium salt, porcine was purchased from MP Biomedicals (China). Cytochalasin D and Hydroxy Dynasore were purchased from Tocris Bioscience (Bristol, United Kingdom). Phenothiazine was purchased from TCI (Portland, OR). Wortmannin was obtained from APExBIO Technology (Boston, MA). Pitstop 2 and filipin III were obtained from Sigma-Aldrich (St. Louis, MO). Methyl-beta-cyclodextrin was bought from Alfa Aesar (Haverhill, MA). Alexa Fluor 555 NHS ester and Alexa Fluor 647 NHS ester were purchased from Life Technologies (Eugene, OR). Ethidium bromide succinimidyl ester (EB-NHS) and thiazole orange succinimidyl ester (TO-NHS) were purchased from Biotium (Fremond, CA). EGFP-Rab5 was a gift from Marci Scidmore (Addgene plasmid # 49888), mEmerald-Caveolin-C-10, mEmerald-Clathrin-15, and mEmerald-Rab7a-7 were gifts from Michael Davidson (Addgene plasmid # 54025, # 54040, and #54244). FIBCD1 Human Tagged ORF Clone plasmid was purchased from Origene (RC206180). The FIBCD1 CRISPR/Cas9 KO plasmid (with GFP tag) and the control CRISPR plasmid were purchased from Santa Cruz Biotechnology (sc-413736, sc-418922) (Santa Cruz, CA). ER-Tracker, Mito-Tracker, LysoTracker, and antibodies (caveolin-1, 3267; anti-rabbit IgG with AF647, 4414; clathrin, 2410) were purchased from Cell Signaling Technology (Danvers, MA).

## METHODS

### *Preparative reversed-phase (RP) high-performance liquid chromatography (HPLC)*

Preparative HPLC was performed on a Thermo Ultimate 3000BX HPLC instrument using a Phenomenex C18 reversed-phase preparative column (Kinetex 5 μm EVO, 250 × 21.2 mm). Nonlinear gradients of 0−100% acetonitrile (with 0.1% TFA) in water (with 0.1% TFA) were employed, and the gradient parameters were adjusted for each product to achieve desired separation efficiencies. A multiwavelength UV−vis detector was used to monitor the absorbance at 215, 280, 480, and 569 nm.

### *Analytical HPLC*

The purity of the peptide was analyzed on a Thermo Ultimate 3000SD HPLC instrument using a Phenomenex C18 reversed-phase analytical column (Kinetex 2.6 μm EVO, 250 × 4.6 mm). A gradient of 0−100% acetonitrile (with 0.1% TFA) in water (with 0.1% TFA) was employed with a flow rate of 1 mL/min. A UV−vis detector was used to monitor the absorbance at 280 or 560 nm. The purity of all cyclic peptides used for binding assays and biological activity assays was >95%.

### *Mass spectrometry*

The MS and MS/MS spectra were obtained using a SCIEX 5800 matrix-assisted laser desorption ionization time-of-flight (MALDI-TOF) mass spectrometer.

### *Solid-phase peptide synthesis*

The peptides were synthesized following the standard Fmoc SPPS coupling process. Unless otherwise noted, Rink Amide MBHA resin was used for the synthesis. To couple amino acids to the resin, the Fmoc group on the resin was first removed by 20% piperidine/DMF solution (10 min, three times). Fmoc-AA-OH (3 equiv), DIEA (5 equiv), and HBTU (2.8 equiv) were mixed in DMF for 10 min, and the solution was then introduced to the deprotected resin. The mixture was gently agitated at room temperature for 1 h, followed by draining and washing (DMF, methanol, and DCM, three times each). To label the peptides with fluorophores, the corresponding dye-COOH was coupled at the N-terminal via a secondary amine linker using the SPPS procedure described above.

For constructing cyclic peptides, Fmoc-propargylglycine-OH (Pra) and Fmoc-azidolysine-OH (Az4) were inserted at the N and C terminals respectively. A Cu-catalyzed click reaction was used for cyclization. Specifically, resins were incubated in 20% lutidine/ DMF with CuI (2.5 equiv) and L-ascorbic acid (5 equiv) at room temperature overnight. After cyclization, the beads were washed with sodium diethyldithiocarbamate (5% w/v) and DIEA (5% v/v) in DMF to remove the copper catalyst.

To cleave peptides off from the resin, a cleavage solution composed of TFA/TIPS/ddH2O (95:2.5:2.5) was used. The crude peptides were purified by preparative RP-HPLC, and the product purity and identity were confirmed by analytical RP-HPLC and mass spectrometry. RB-cy(YYTYT), $C_{79}H_{97}N_{14}O_{15}$+, $[M + H]^+$ calculated 1481.73, found 1481.73. RB-cy(TYYTY), $C_{79}H_{97}N_{14}O_{15}^+$, $[M + H]^+$ calculated 1481.73, found 1481.73. RB-cy(YTYYT), $C_{79}H_{97}N_{14}O_{15}^+$, $[M + H]^+$ calculated 1481.73, found 1481.78. RB-cy(YYYYY), $C_{89}H_{101}N_{14}O_{15}^+$, $[M + H]^+$ calculated 1605.76, found 1606.80. RB-cy(TTTTT), $C_{64}H_{91}N_{14}O_{15}^+$, $[M + H]^+$ calculated 1295.68, found 1295.81. RB-cy(YSYYS), $C_{77}H_{93}N_{14}O_{15}^+$, $[M + H]^+$ calculated 1453.69, found 1453.77. RB-cy(SYYSY), $C_{77}H_{93}N_{14}O_{15}^+$, $[M + H]^+$ calculated 1453.69, found 1453.73. RB-TAT$_{47\text{-}57}$, $C_{97}H_{157}N_{36}O_{16}^+$, $[M + H]^+$ calculated 2082.26, found 2083.51. RB-cTAT, $C_{94}H_{157}N_{36}O_{15}^+$, $[M]^+$ calculated 2030.26, found 2030.62. AF555-cy(YSYYS), $C_{71}H_{76}N_{14}O_{23}S_2^{2-}$, $[M + H]^+$ calculated 1556.47, found 1556.34. AF647-cy(YSYYS), $C_{89}H_{113}N_{14}O_{26}S_4^{3-}$, $[M + H]^+$ calculated 1921.68, found 1921.67. Fluo-cy(YSYYS), $C_{69}H_{72}N_{12}O_{19}$, $[M + H]^+$ calculated 1372.50, found 1372.25.

***Cell lines and cell culture***

The human glioblastoma cell line (U87) was purchased from ATCC. The human embryonic kidney cell line (HEK-293T), the human osteosarcoma cell line (U2OS), the human breast cancer cell line (MCF-7), the human metastatic melanoma cell lines (IGR-37, IGR-39 and WM266-4) and the human cervical carcinoma cell line (HeLa) were gifted by Prof. Yinsheng Wang (UC Riverside). The non-tumorigenic epithelial cell line (MCF 10A) and the human breast adenocarcinoma cell line (MDA-MB-231) were gifted by Prof. Wenwan Zhong (UC Riverside). The canine epithelial kidney cell line (MDCK) and the African green monkey kidney cell line (Vero) were gifted by Prof. Hai Rong (UC Riverside). The human colon carcinoma cell line (HCT116) was gifted by Prof. Xuan Liu (UC Riverside). The human liver carcinoma cell line (HEPG2), the rat liver cell line (MCA7777), and the mouse sarcoma cell line (J774A.1) were gifted by Prof. Joseph Genereux (UC Riverside).

For adherent cell lines, cells were cultured in Dulbecco's modified Eagle's medium (DMEM, Corning) supplemented with 10% heat-inactivated fetal bovine serum (FBS, Gibco) and 100 U/mL penicillin/streptomycin (Sigma). Cells were cultured with 5% $CO_2$ in a 37 °C incubator. A trypsin-EDTA solution (0.05%, Sigma) was used for passaging once the cells reached 80−90% confluency.

For suspension cell lines, cells were cultured in RPMI 1640, 1× (Corning) medium supplemented with 10% heat-inactivated fetal bovine serum (Gibco) and 100 U/mL penicillin/streptomycin (Sigma). Cells were cultured with 5% $CO_2$ in a 37 °C incubator. Fresh culture media was used to dilute the original media at a 1:5 ratio after every two doubling cycles.

***Confocal imaging experiments***

To image the cells, a Zeiss 880 inverted confocal laser scanning microscope (Carl Zeiss, Germany) was used. Image acquisition and analyses were carried out using the manufacturer's software (ZEN, Carl Zeiss). Quantification of fluorescence intensity in single cells was performed using Fiji software.

***Flow cytometry experiments***

To perform flow cytometry experiments, a NovoCyte flow cytometer (NovoExpress) was used. Quantification of fluorescence intensity was achieved using the Novoxpress software. Fifty thousand cells were analyzed for each condition. A green fluorescent dye used for DNA staining, YOYO, was included in all the flow cytometry experiments, serving as a cell live/dead indicator. For the YOYO-only group, the cells were treated with trypsin for 5-10 min and subsequently fixed in a 1.5 mL centrifuge tube using 4% paraformaldehyde at room temperature for 15 min. Then, fixed cells were washed with PBS once and were permeabilized using 90% MeOH on ice for 15 min. For all conditions, cells were incubated with YOYO in a phenol-free medium for half an hour, and then were centrifuged to remove the YOYO solution and resuspended in a fresh phenol-free medium before performing flow cytometry.

***Dye-EPP incubation***

For general incubation, 300 k U87 cells were seeded in a 35×10 mm culture dish and incubated overnight under normal culturing conditions. Stock solutions of Dye-EPPs were made in DMSO to reach a concentration of 500 µM. Fresh cell culture medium was used to dilute the stock solution to 500 nM. Cells were incubated with RB-EPPs for desired time periods, followed by a one-time wash with phenol-free fresh culture medium before measurements.

***Octanol-water partition assay***

For each RB-EPP, 250 µL of octanol and 250 µL of water were added to a 1.5 mL centrifuge tube. 2 µL of RB-EPP (in DMSO) was added to the solution at a concentration of 500 µM. After vortexing, the mixture was centrifuged at 21,000 G for 10 min to separate the octanol and water phases. Samples were collected from both phases and quantified by Analytical HPLC separately.

***Concentration-dependent uptake experiment***

U87 cells were incubated with RB-EPP6 at different concentrations (20 nM, 200 nM, 2 µM, 20 µM, 50 µM, and 100 µM). After washing with fresh medium once cells were examined by confocal imaging.

***Time-dependent uptake experiment***

U87 cells were incubated with RB-EPP6 at 500 nM for 1 h. After washing with fresh medium once, the cells were kept in the 5% $CO_2$ incubator at 37 °C for desired time durations before confocal imaging.

***Temperature-dependent uptake experiment***

U87 cells were kept in a 4 °C cold room for half an hour. After that, cells were incubated with RB-EPP 6 at 500 nM at 4 °C. Cells in the control group were maintained and incubated at 37 °C. After 1 h, cells were washed with fresh medium once before confocal imaging.

***PAMPA assay***

All the RB-EPPs were diluted in PBS to 100 μM. 700 μL of the testing peptides were added to the apical chamber of a Corning BioCoat Pre-coated PAMPA Plate. 200 μL PBS was added to the basolateral chamber. The plate was kept at 37 °C with 5% $CO_2$ for 5 h, after which 80 μL samples were collected from each chamber for RB fluorescence intensity reading using the Synergy H1 microplate reader.

***Endocytosis inhibitors treatment***

U87 cells were incubated with different endocytosis inhibitors for 1 h. Then, the media was replaced with fresh media containing 500 nM of RB-EPP6. After 1 h, a trypsin-EDTA solution (0.05%, Sigma) was added to detach the cells. The cells were collected by centrifugation and resuspended in phenol-free media. Once resuspended, cells were filtered into glass tubes for flow cytometry analyses.

***Resazurin assay***

Resazurin was dissolved in DI water at a stock concentration of 6 mg/mL. After cell treatments, 40 μL resazurin solution at 60 μg/mL diluted with cell culture media was added to each well containing 200 μL cell culture media in a 96-well plate. The fluorescence intensity of the samples was recorded by a plate reader at 532 nm excitation and 585 nm emission wavelengths.

***Organelle tracker colocalization experiment***

Stock solutions of trackers for mitochondria, lysosome, and ER were diluted with cell culture medium into 100 nM, 500 nM, and 4 μM, respectively. RB-EPP6 was added to each working solution at 500 nM. U87 cells were incubated with the trackers and RB-EPP6 for 1 h and then washed once before confocal imaging.

### *Plasmid transfection*

Cells were seeded in either a 35x10 mm dish (300k per well) or in a 96-well plate (10k per well) for overnight culture. Plasmids (caveolin, 75 ng; clathrin, 100 ng; rab5, 100 ng; rab7a, 100 ng for every 300k cells; *FIBCD1* ORF clone plasmid, 100 ng and 1 μg; Control CRISPR, 200 ng, *FIBCD1* CRISPR/Cas9 KO, 200 ng; Ctrl siRNA, 3 pmol; *CAV1* siRNA, 3 pmol; *CLTC* siRNA, 3 pmol for every 10k cells) were diluted with Plasmid Transfection Medium (sc-108062). UltraCruz Transfection Reagent (Santa Cruz Biotechnology) or Lipofectamine Reagent (Invitrogen) was also diluted with Plasmid Transfection Medium. Plasmids and transfection reagent were mixed well and kept at room temperature for 20 min. The media was replaced with a PS-free medium and the mixture was added to the cells for another 24 h culture.

### *Immunostaining assay*

Cells were fixed with ice-cold 90% MeOH for 10 min on ice, followed by 1x PBS wash for 5 min three times. 5% milk in PBS was used to block the cells for an hour at room temperature. The primary antibodies for the proteins of interest were diluted into working concentration using 0.5% milk in PBS. The cells were incubated with primary antibody at 4 °C overnight. The cells were washed three times with 1x PBS for 5 min. The secondary antibody conjugated with Alexa Fluor 647 was diluted in 0.5% milk in PBS into a working concentration. The cells were incubated with a secondary antibody for an hour at room temperature, followed by 1× PBS wash for 5 min three times.

### *Heparin-binding assay*

U87 cells were treated with heparin sulfate at 10, 20, 50, 100, and 200 μg/mL in a 5% $CO_2$ incubator at 37 °C for 30 min. Afterward, the cell medium was replaced by a medium with both heparin sulfate and 500 nM RB-EPP6 for another 1 h incubation. A trypsin-EDTA solution (0.05%, Sigma) was added to detach the cells. The cells were collected by centrifugation. Fresh phenol-free medium was added to each condition. Once resuspended, cells were filtered into glass tubes before flow cytometry.

### *RNA-seq studies*

In a 100×15 mm dish, 1 million U87 cells were seeded for overnight culture in a full DMEM medium containing 10% FBS and 1% PS. RB-EPP6 diluted with fresh culture medium at 500 nM was used to replace the old medium the next day. After 1 h incubation in the 5% $CO_2$ incubator at 37 °C, the cells were treated with trypsin and resuspended in a fresh phenol-free medium after centrifuge. Then, cells were filtered into glass tubes for fluorescence-activated cell sorting (FACS). Cell populations with the highest and lowest 30% fluorescence intensity were collected separately. RNA extraction was performed using the RNeasy Micro Kit (QIAGEN) right after the FACS

experiment. Poly-A selective RNA-seq libraries were prepared using the NEB NextUltra II kit (New England Biolabs) and sequenced on an Illumina NextSeq500 with 2×75 pair-end reads. The obtained sequencing data was preprocessed using fastp, aligned using STAR, and counted using featureCounts. Differentially expressed genes were identified using DESeq2. The sequence data and analysis reports are deposited in the GEO repository (Accession # GSE202764)

### *FIBCD1 competition assay*

U87 cells were incubated with 500 nM RB-EPP6 and YOYO, together with chondroitin sulfate (CSA) in complete media. After 1 h, trypsin was used to detach the cells. Cell pellets were collected by centrifugation. Fresh phenol-free medium was added to each condition. Once resuspended, cells were filtered into glass tubes for flow cytometry.

### *Expression and purification of full-length FIBCD1*

Human *FIBCD1* cDNA with an MYC-DDK tag was ordered from Origene (#RC206180) and was expressed using the mammalian expression system kit ExpiCHO$^{TM}$ (#A29133) as generally described by MAN0014337. Expression of FIBCD1 occurred for 8 days while incubating the cells at 37 °C with 5% $CO_2$. MYC-DDK-tagged FIBCD1 was extracted utilizing the Mem-PER™ Plus membrane protein extraction kit (#89842) as described by MAN0011863. Purification was first performed using anti-DYKDDDDK G1 affinity resin (#L00432-10). MYC-DDK-tagged FIBCD1 was eluted using a competitive binding method with 1 mg/mL FLAG peptide, followed by a HiLoad 16/600 S-200 pg (Cytiva) column separation. 1xPBST (137 mM NaCl, 3 mM KCl, 8 mM $Na_2HPO_4$, 1.5 mM $KH_2PO_4$, 0.06% Tween 20, pH 7.4) was used for purification. The obtained full-length FIBCD1 was quantified using BCA.

### *Fluorescence polarization (FP) assay*

Solutions of RB-EPPs (in 1% BSA/PBST) were added to different concentrations of full-length FIBCD1 (in 1% BSA/PBST). The final concentration of RB-EPPs was 50 nM. The solutions were incubated at room temperature for 30 minutes on an orbital shaker, and the fluorescence polarization values were quantified using a BioTek Synergy H1 plate reader, with an FP filter set (excitation 530/25, emission 590/35). The resulted FP values were then plotted and fitted with a Hill function using OriginPro.

### *FIBCD1-KO CRISPR in Cas9-U87*

The cas9-expressing stable U87 cell line and three *FIBCD1*-sgRNA CRISPR Lentivirus (different sgRNA constructs) were purchased from ABM (#205521110101). The Cas9-U87 cell line was allowed to grow to about 70% confluency in a 100 mm petri dish in complete DMEM. These cells were then passaged into a 24-well culture plate at approximately 50,000 cells per well for each

*FIBCD1* Lentiviral target, at different multiplicities of infection (MOI) levels. The cells were then incubated at 37 °C and 5% $CO_2$. After 48 hours, the media was removed and replaced with fresh new media. Cell selection was carried out using G418 (Alfa Aesar) at 1 mg/mL for 7 days, and the surviving cells were collected and analyzed for *FIBCD*-KO efficiency by western blot. The best candidate was used for the RB-EPP6 uptake experiments described above.

### *MDCK transwell assay*

200k MDCK cells were seeded in 200 µL of complete media in the apical side transwell insert (12-well 8 µm pore size Transwell-65 mm). In addition, 1 mL of complete growth media is added to the basolateral chamber. Six transwell inserts were prepared for a TAT peptide labeled with RB (RB-$TAT_{47-47}$), and six other inserts were prepared for RB-EPP6. The plate was incubated for 2-3 days at 37 °C with 5% $CO_2$. The electrical resistance of the MDCK transwell inserts was measured using the EVOM Epithelial Voltohmmeter to monitor the integrity of tight junctions for the following two days. On day 3, serum-free media was used to wash both the apical (200 µL) and basolateral (1 mL) wells three times. Cells were kept in a 37 °C/5% $CO_2$ cell incubator equilibrating for 15 min.

The assay was carried out in apical to basolateral directions. Both RB-$TAT_{47-47}$ and RB-EPP6 were prepared at 10 µM in serum-free media. The apical chamber was replaced with 200 µL testing compounds. The plate was incubated in the cell culture incubator for 3 h. After that, 80 µL of the samples were collected from each chamber for RB fluorescence intensity reading using a Synergy H1 microplate reader.

### *Radiosynthesis of $^{18}$F-2 and $^{18}$F-EPP6*

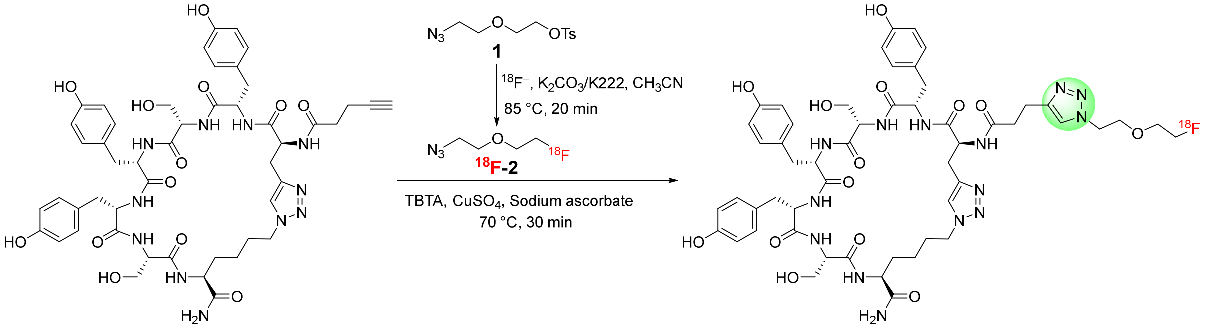


$^{18}$F-EPP6

[$^{18}$O]$H_2O$ was purchased from Huayi Isotopes Co. Other chemicals and solvents were obtained from Sigma-Aldrich unless otherwise noted. The QMA ion exchange cartridges were purchased from ABX advanced biochemical compounds GmbH (Germany). Analytical high-performance liquid chromatography (HPLC) was accomplished with a Thermo Fisher Scientific UltiMate 3000 HPLC System using a reversed-phase C18 column (250 × 4.6 mm, 5 µm). The HPLC system was operated by Chromeleon 7.2 software. The flow rate was 1 mL/min with gradient mobile phase of acetonitrile (MeCN) and water with 0.1% trifluoroacetic acid (TFA): 0 – 12.5 min, 0 – 80% MeCN;

12.5 – 13.0 min, 80% MeCN; 13.0 - 13.5 min, 80 - 0% MeCN; 13.5 – 15 min, 0% MeCN. The UV absorbance was monitored at 214 nm. Semi-preparative HPLC purification was performed using a Phenomenex Luna C18(2) reversed-phase column (250 × 10 mm, 5 μm). The UV absorbance was monitored at 214 nm. Semi-preparative HPLC purification was performed using a Phenomenex Luna C18(2) reversed-phase column (250 × 10 mm, 5 μm). The flow rate was 3.5 mL/min with gradient mobile phase of MeCN and water for $^{18}$F-**2** purification: 0 – 3 min, 0% MeCN; 3 – 30 min, 0 – 75% MeCN; 30 – 32.5 min, 75% MeCN; 32.5 – 33.5 min, 75 – 0% MeCN; 33.5 – 40 min, 0% MeCN; and with gradient mobile phase of MeCN and water with 0.1% TFA for $^{18}$F-EPP6 purification: 0 – 3 min, 0% MeCN; 3 – 30 min, 0 – 75% MeCN; 30 – 32.5 min, 75% MeCN; 32.5 – 33.5 min, 75 – 0% MeCN; 33.5 – 40 min, 0% MeCN.

The [$^{18}$F]fluoride ion was produced by the nuclear reaction [$^{18}$O](p, n) [$^{18}$F] on a GE PETtrace 800 cyclotron. [$^{18}$F]fluoride (approximate 37 GBq) in [$^{18}$O]water was passed through an anion exchange QMA cartridge preconditioned with 10 mL of ethanol and DI-water, respectively. The retained [$^{18}$F]fluoride was eluted to a V-vial with a potassium bicarbonate solution (0.4 M in DI-water, 0.4 mL). A solution of Kryptofix 222 (15 mg in 1.0 mL anhydrous MeCN) was added to the V-vial and then dried at 100 °C with nitrogen gas flow. MeCN was added to the V-vial for azeotropic drying. The precursor 2-(2-azidoethoxy) ethyl 4-methylbenzenesulfonate (**1**) (15.0 mg in 0.8 mL anhydrous MeCN) was added to the V-vial and heated at 85 °C for 20 min. The crude reaction mixture was then analyzed by analytical HPLC and purified by semi-preparative HPLC. The EPP6 peptide acetylene (1.2 mg, 1.2 μmol), tris[(1-benzyl-1H-1,2,3-triazol-4-yl)methyl] amine (TBTA, 20.0 mg, 37.7 μmol) in 0.25 mL $CuSO_4$ (1.0 M) and sodium ascorbate (40.0 mg, 0.2 mmol) in 0.1 mL $H_2O$ were added to $^{18}$F-**2**. Subsequently, the reaction was heated at 70 °C for 30 min. the crude reaction mixture was analyzed by analytical HPLC and purified by semi-preparative HPLC to provide the final product, $^{18}$F-EPP6. The total synthesis time was about 180 min, including the HPLC purification time. The specific activity of $^{18}$F-EPP6 was estimated to be 206 GBq/μmol.

***Partition coefficient***

The 1-octanol–phosphate-buffered saline (PBS) partition coefficient was measured at room temperature, and the value was designated as Log P. $^{18}$F-EPP6 (~370 KBq) in 10 μL of PBS (pH = 7.4) was added to an Eppendorf tube containing 500 μL of PBS (pH 7.4) and 500 μL of 1-octanol. The mixture was vortexed for 3 min and then centrifuged (12,500 rpm) for 10 min. The PBS and 1-octanol layers (150 μL of each layer) were pipetted into gamma-counter test tubes, respectively. The radioactivity was determined using a PerkinElmer 2480 WIZARD automatic gamma counter (PerkinElmer Inc., Waltham, MA). The partition coefficient of 1-octanol-to-PBS was calculated as Log P = log([organic-phase cpm]/[aqueous-phase cpm]). Measurements were carried out in quintuplicate. The Log P of $^{18}$F-EPP6 was determined to be –1.25 ± 0.01.

***Mouse serum stability***

Approximately 7.4 MBq of $^{18}$F-EPP6 in 250 μL mouse serum was shaken at 37 °C for 1 or 2 h, after which 10 μL TFA was added. The mixture was vortexed for 3 min and centrifuged for 10 min (12,500 rpm). The supernatant was analyzed by HPLC. Measurements were performed in duplicate.

***Animals***

Animal procedures were approved by the Institutional Animal Care and Use Committee of the University of Southern California. The athymic nude mice were purchased from The Jackson Laboratory (Bar Harbor, ME, USA).

***In vivo microPET imaging***

MicroPET scans were performed using a rodent scanner (Siemens Inveon microPET scanner, Siemens Medical Solutions). Under anesthesia, about 11.1 MBq of $^{18}$F-EPP6 was either intravenously injected into nude mice through the tail vein or administrated by oral gavage. Five-min static scans were performed at 5, 15, 30, and 60 min post-injection (pi) for the intravenous injection, whereas 5-min static scans were performed at 15, 30, and 60 min pi for the administration by oral gavage. A 25-min dynamic PET scan was also carried out for the intravenous injection. The PET data were reconstructed by the 3-dimensional ordered-subsets expectation maximum (3D-OSEM) algorithm. Regions of interest were drawn over normal tissues/organs, such as muscle, liver, and kidneys, on the decay-corrected whole-body coronal images.

**Fig. S1.** (**A-P**) HPLC and MALDI characterization of synthesized peptides.

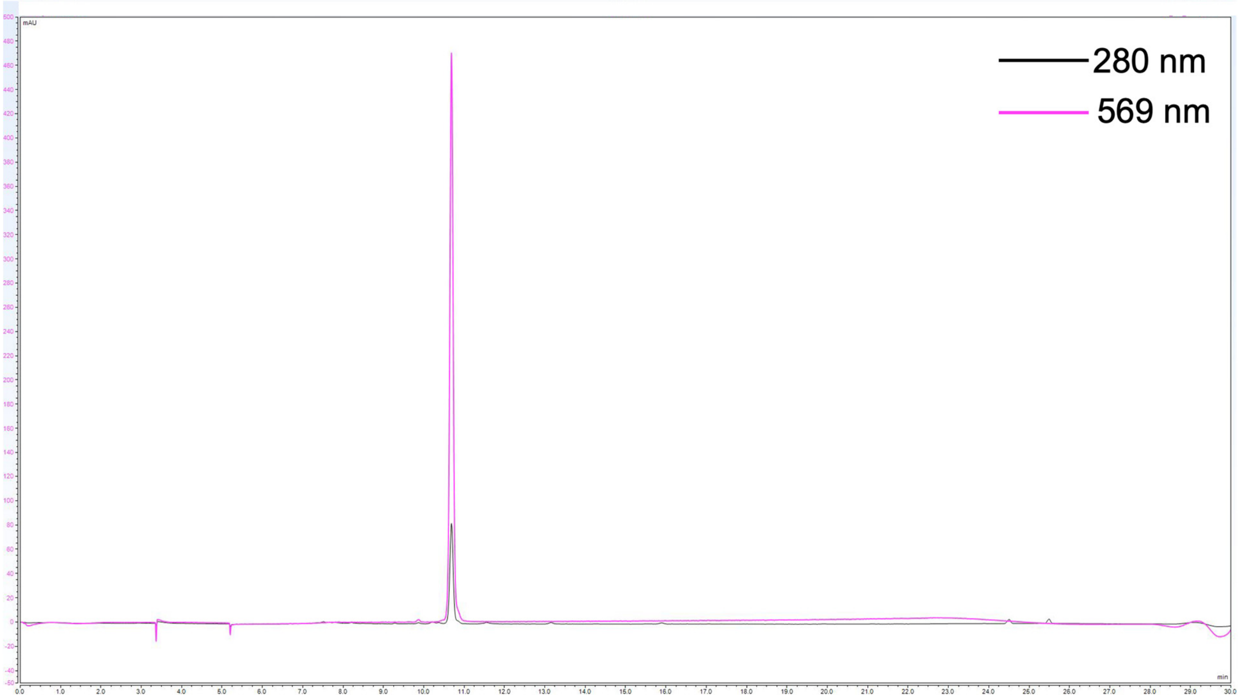


(**A**) HPLC chromatogram of RB-EPP1.

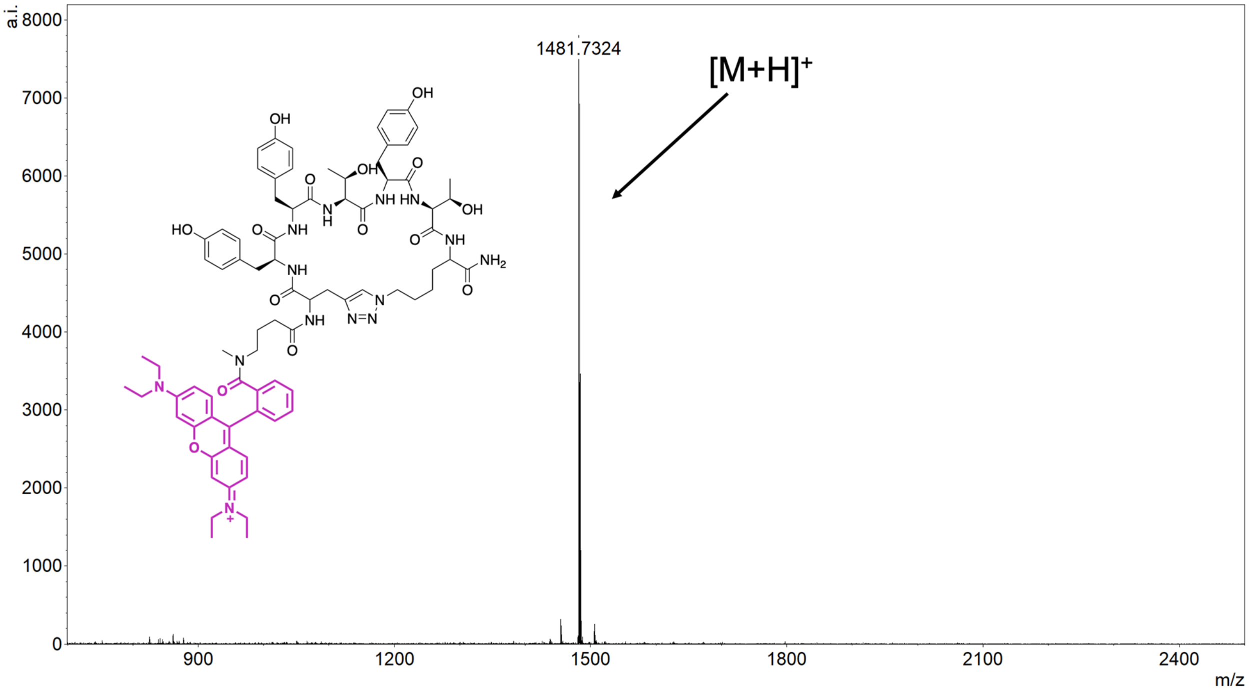


(**B**) Mass spectrum of RB-EPP1.

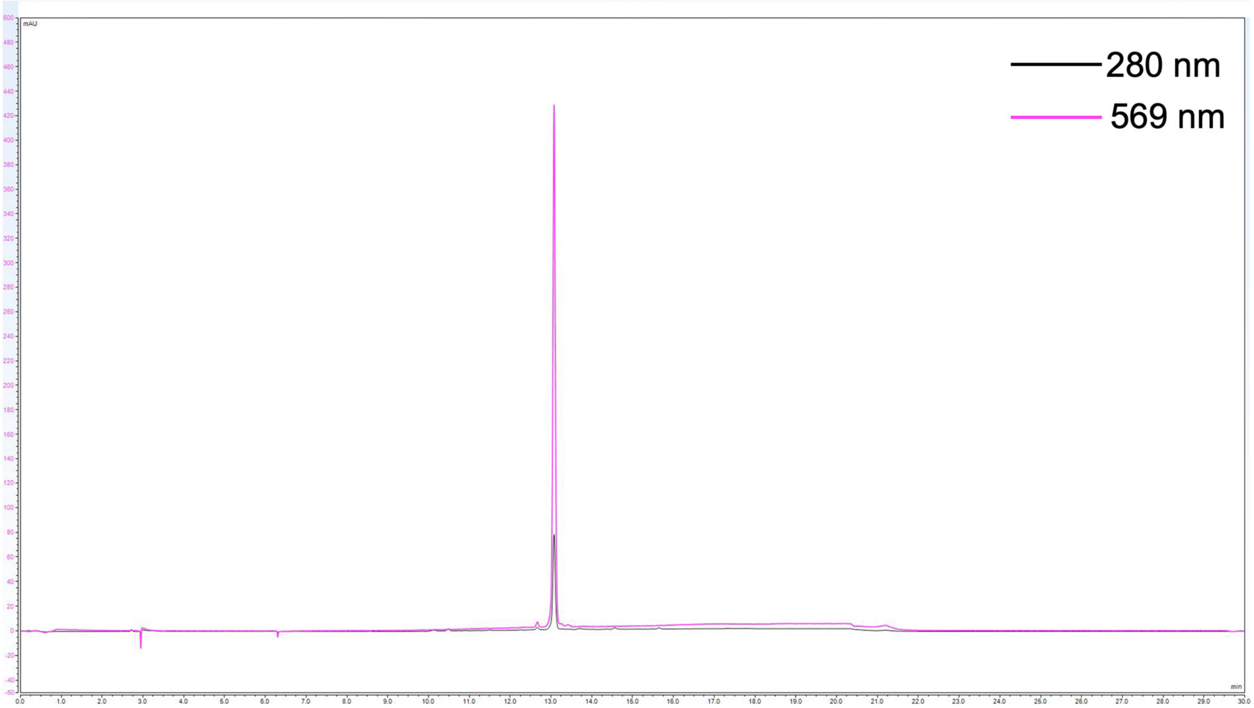


(**C**) HPLC chromatogram of RB-EPP2.

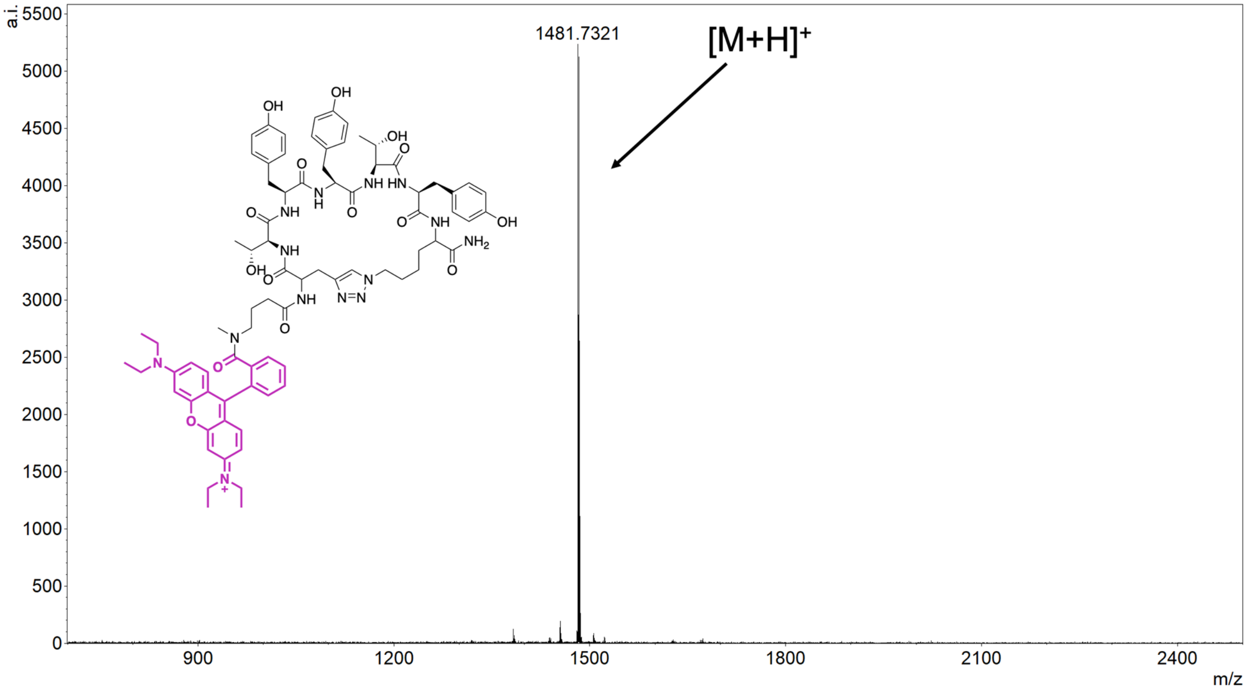


(**D**) Mass spectrum of RB-EPP2.

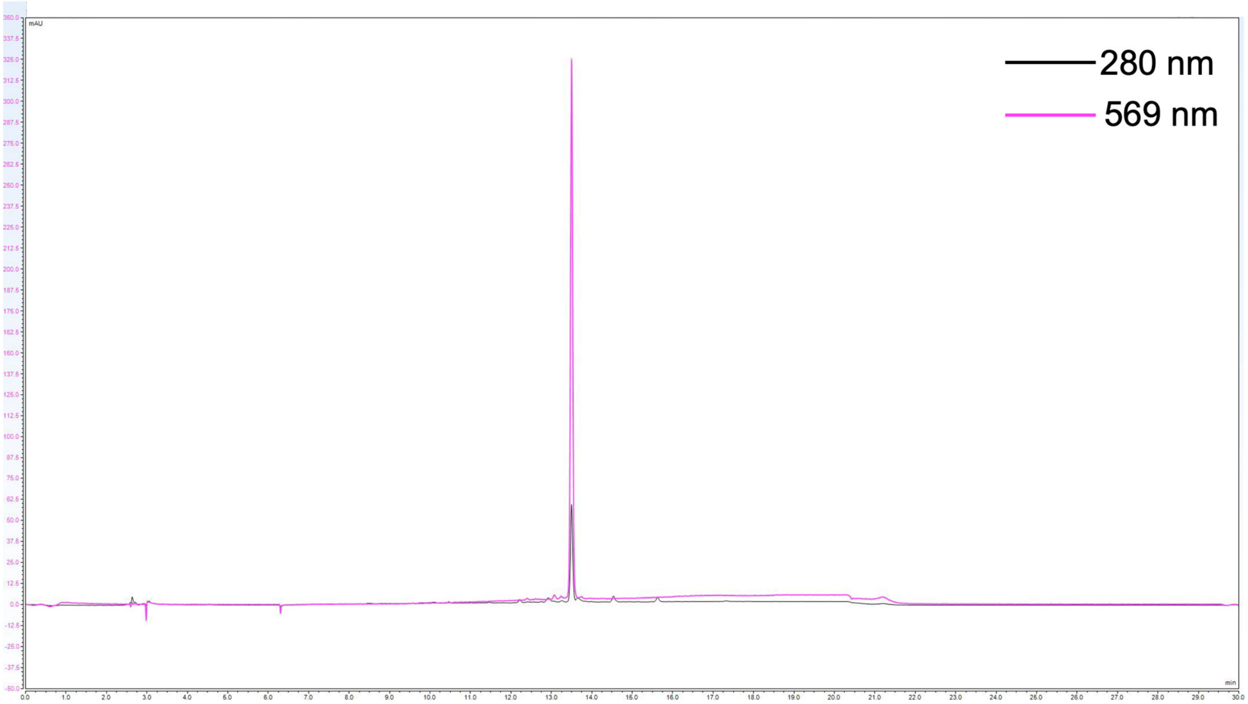


(**E**) HPLC chromatogram of RB-EPP3.

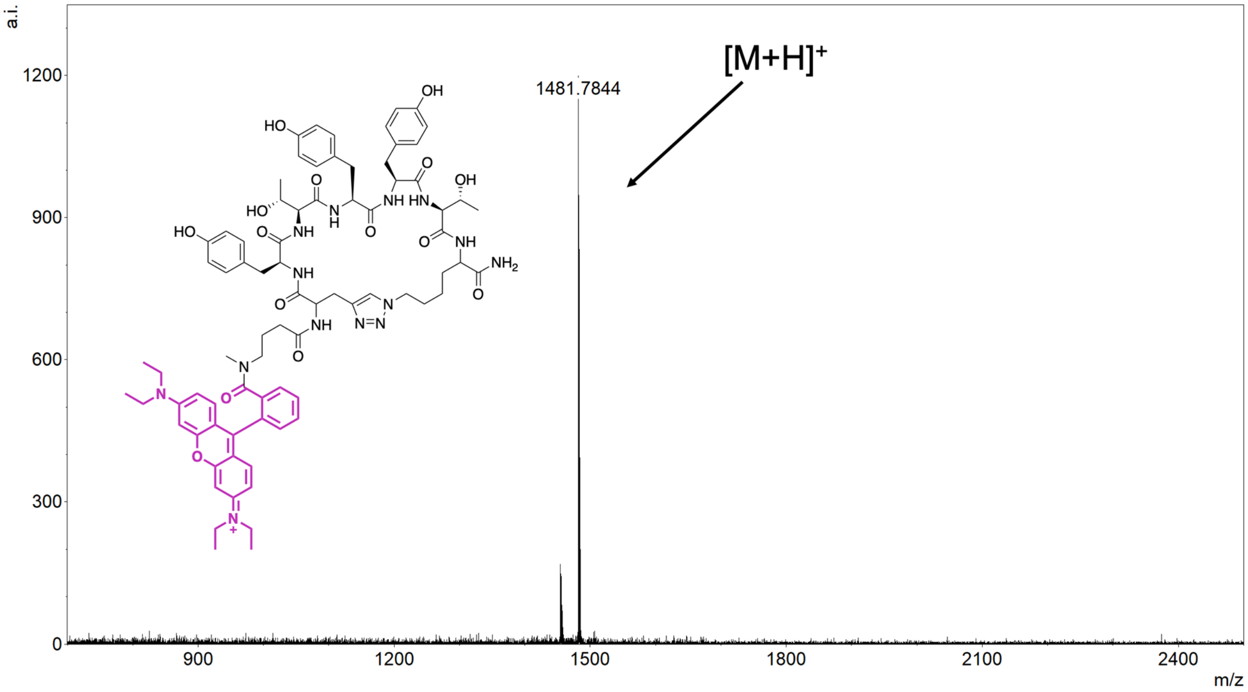


(**F**) Mass spectrum of RB-EPP3.

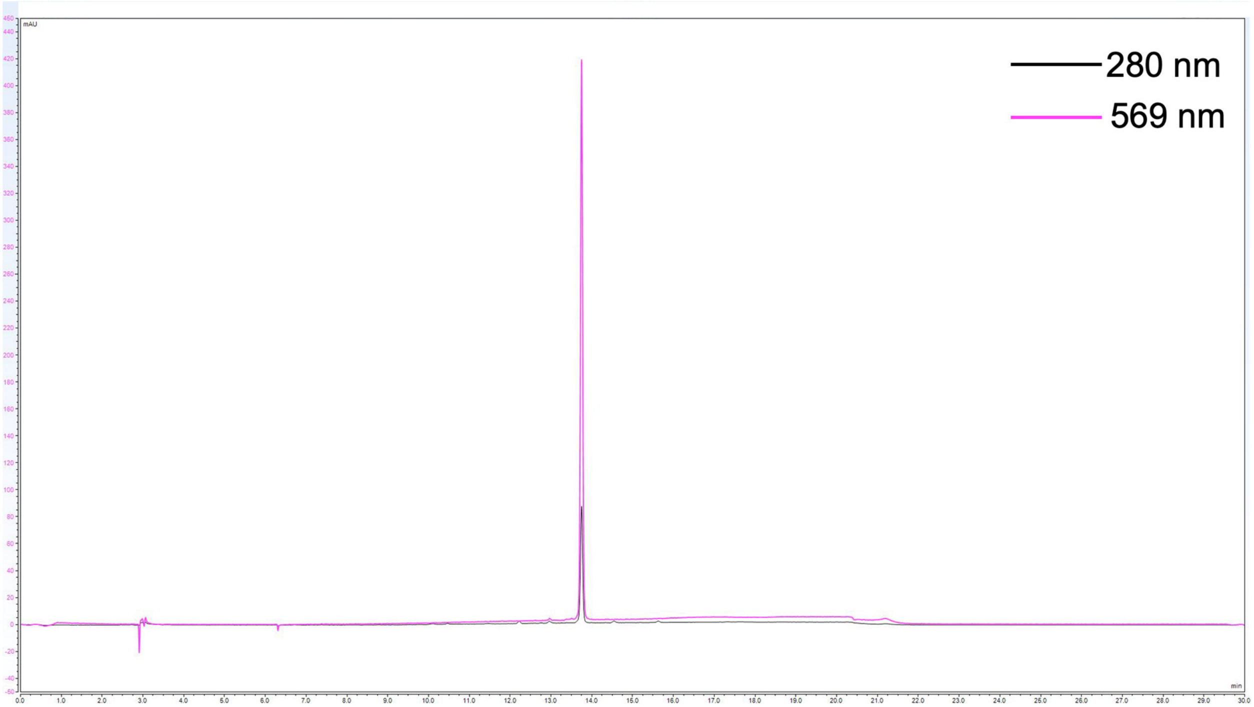

(**G**) HPLC chromatogram of RB-EPP4.

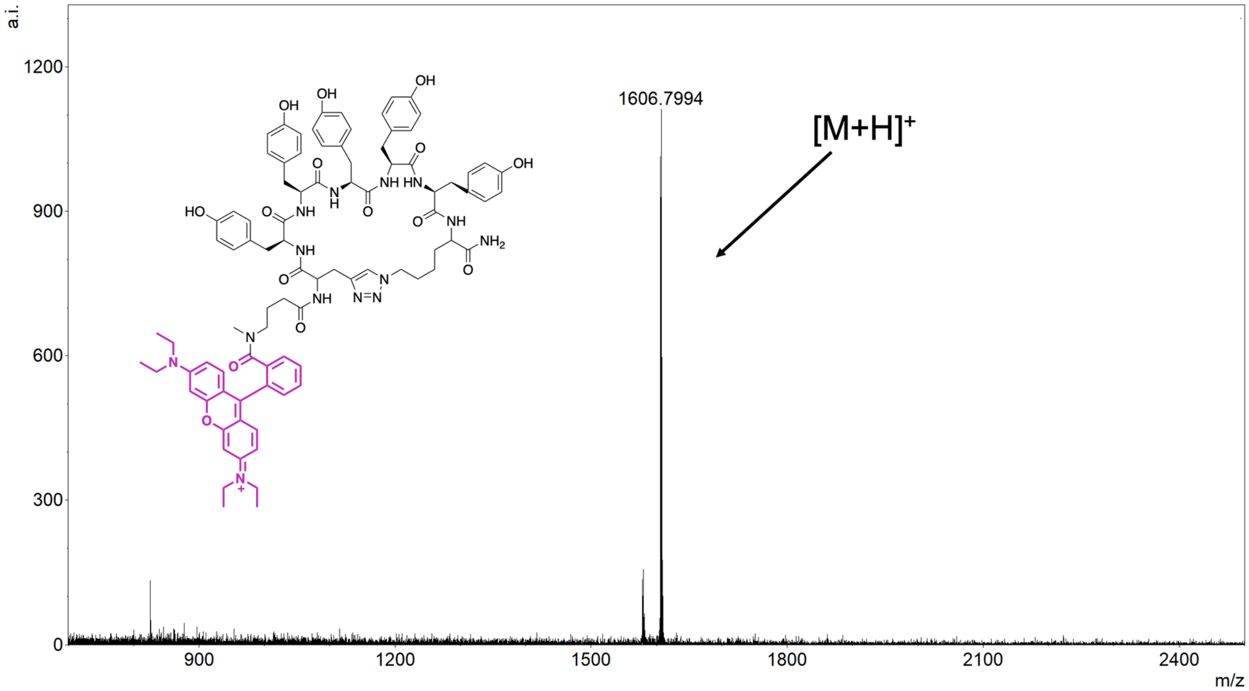

(**H**) Mass spectrum of RB-EPP4.

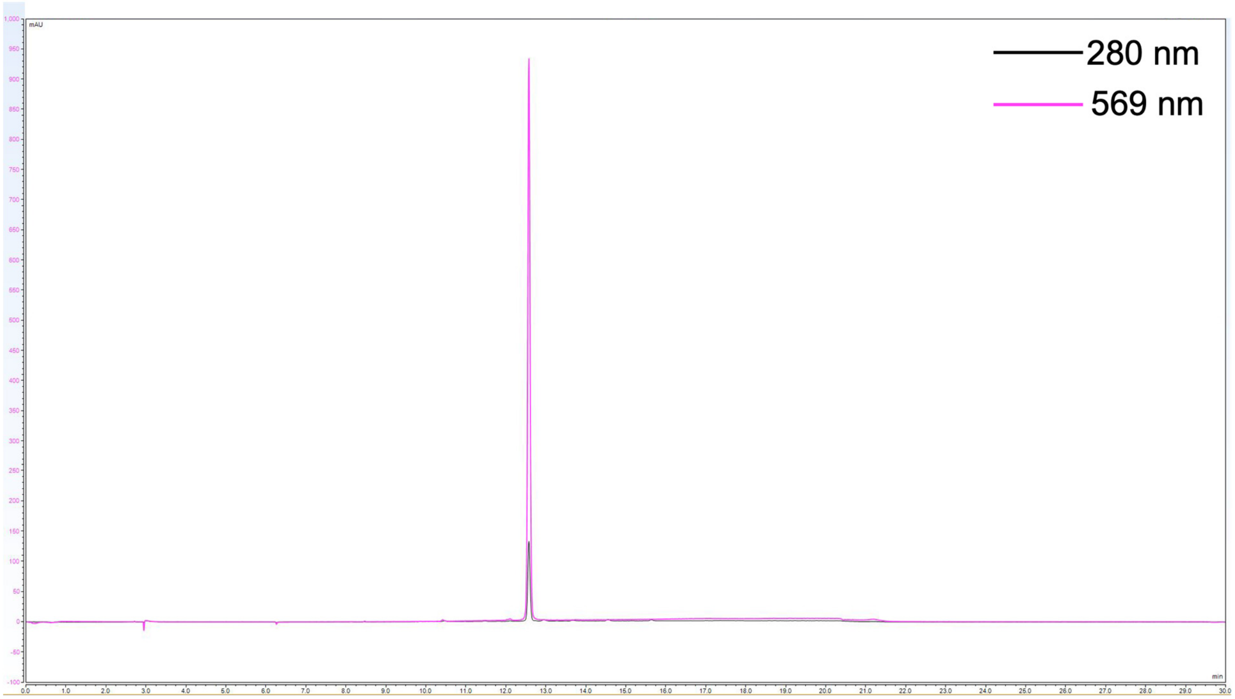


(**I**) HPLC chromatogram of RB-EPP5.

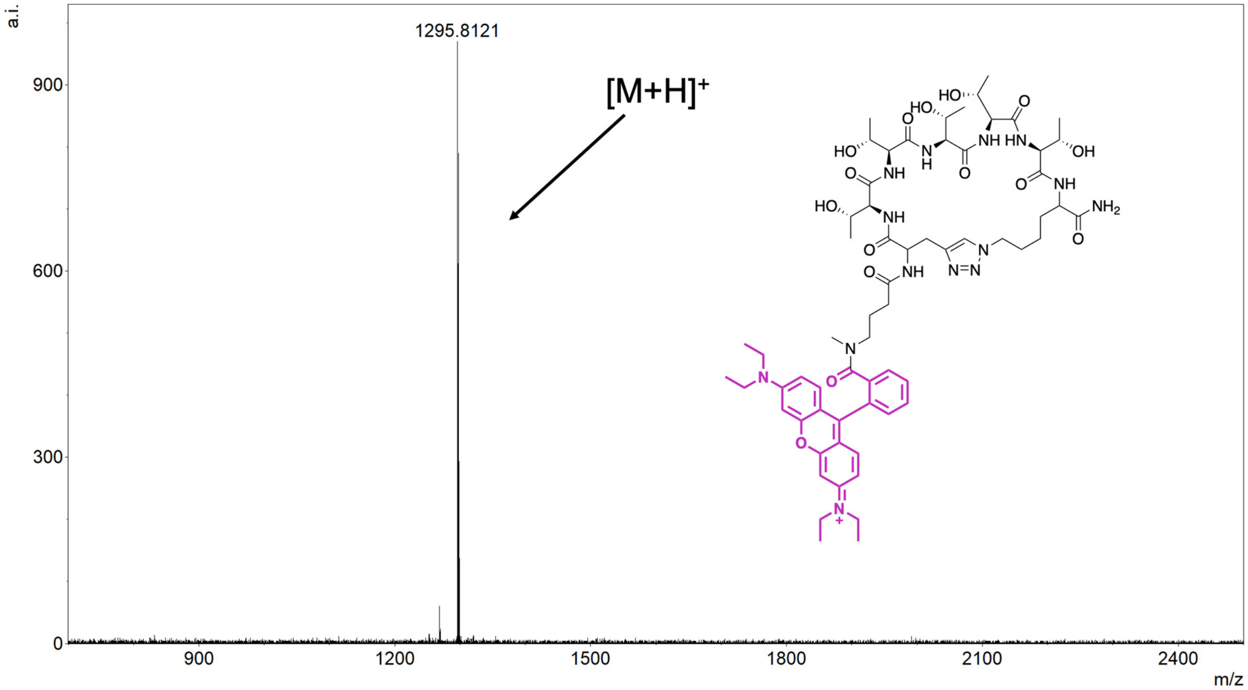


(**J**) Mass spectrum of RB-EPP5.

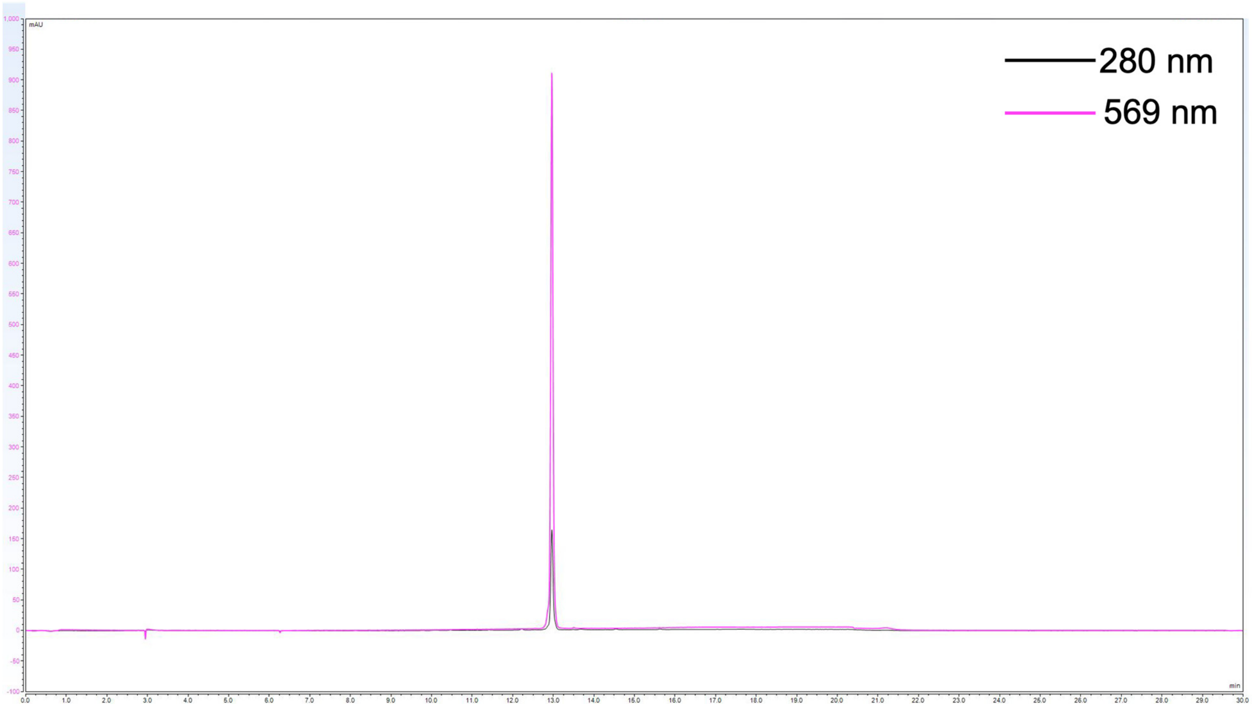


(**K**) HPLC chromatogram of RB-EPP6.

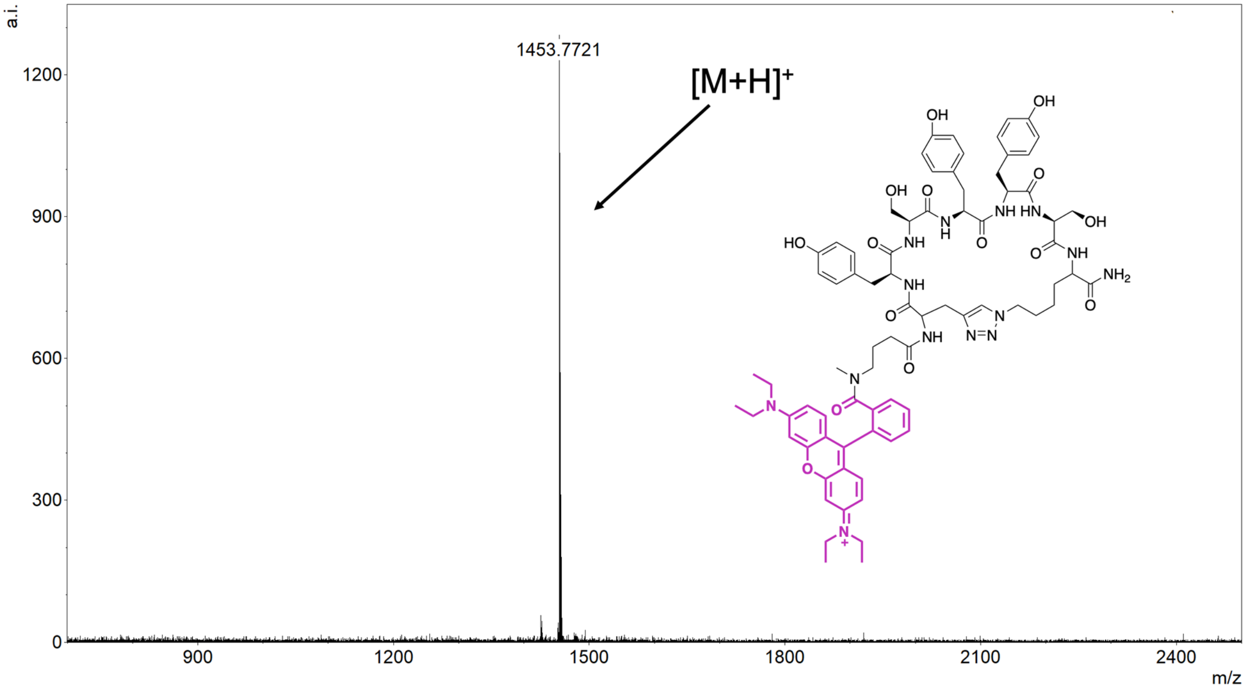


(**L**) Mass spectrum of RB-EPP6.

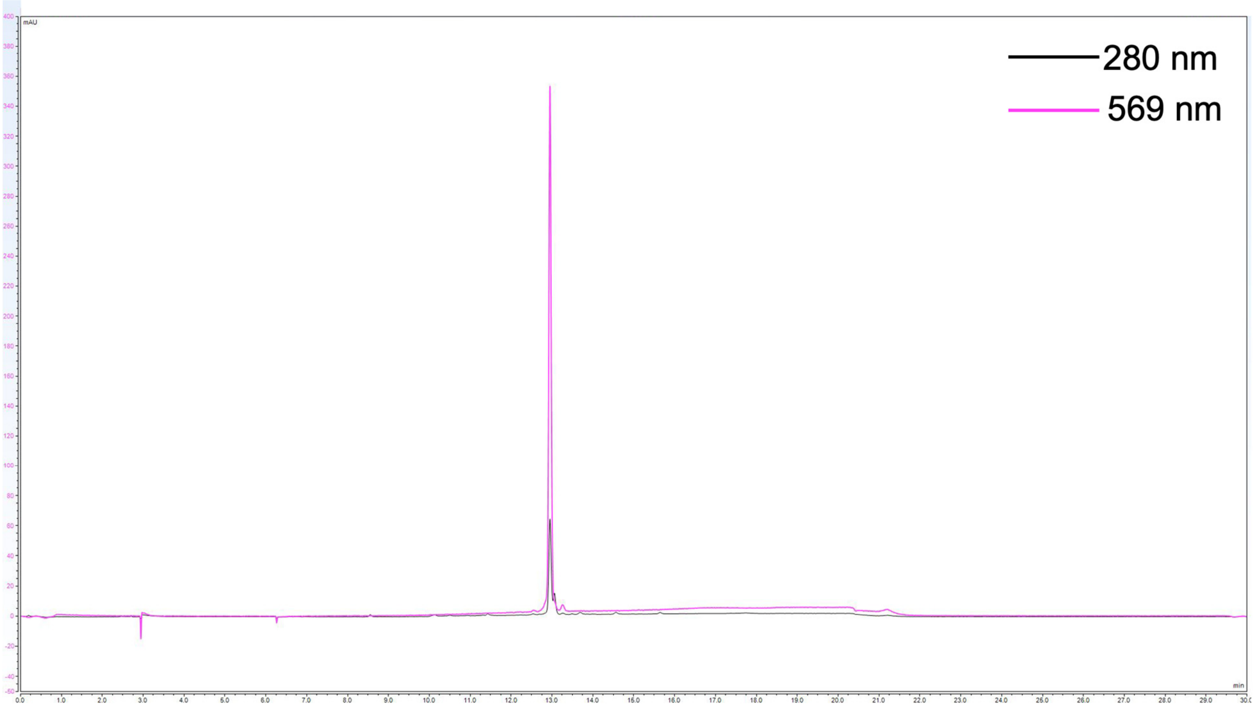


(**M**) HPLC chromatogram of RB-EPP7.

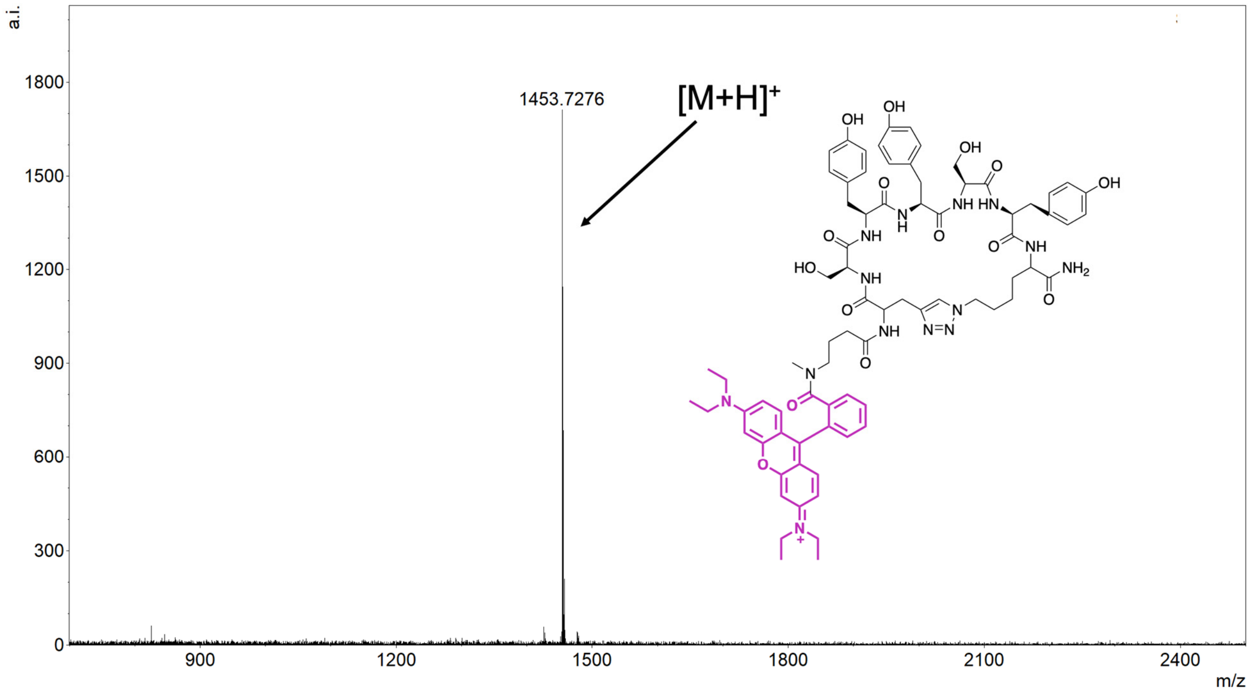


(**N**) Mass spectrum of RB-EPP7.

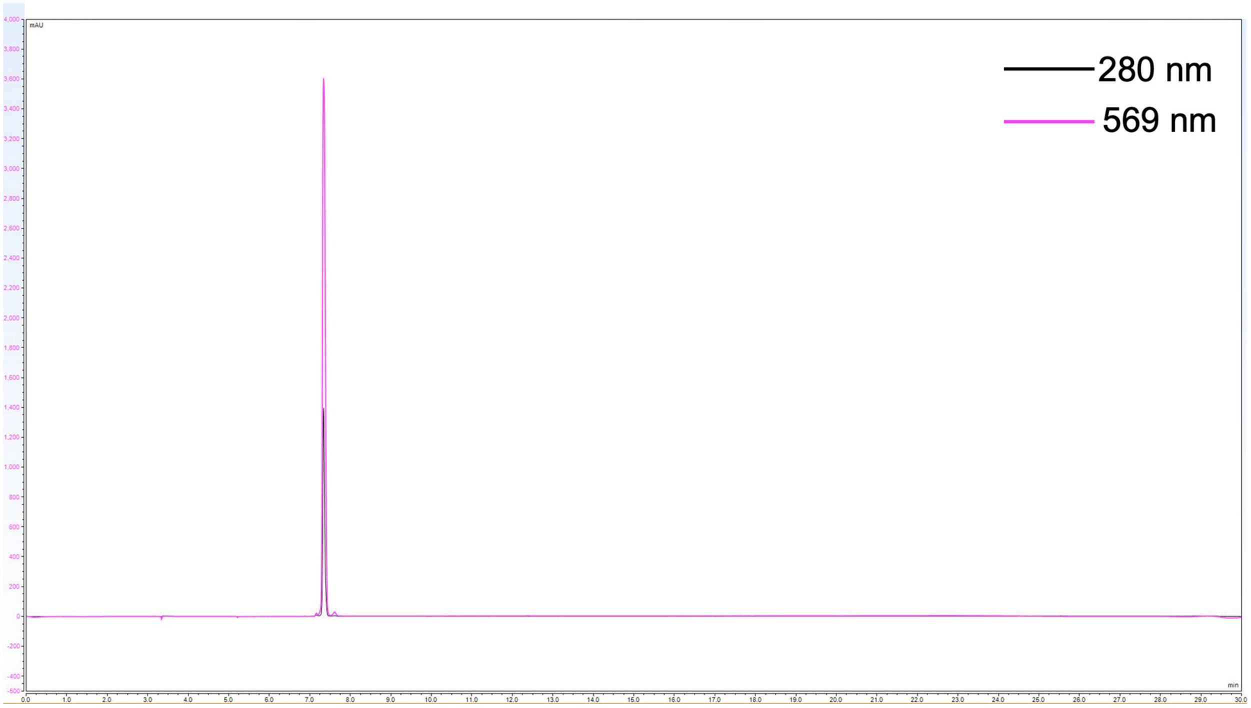


(**O**) HPLC chromatogram of RB-TAT47-57.

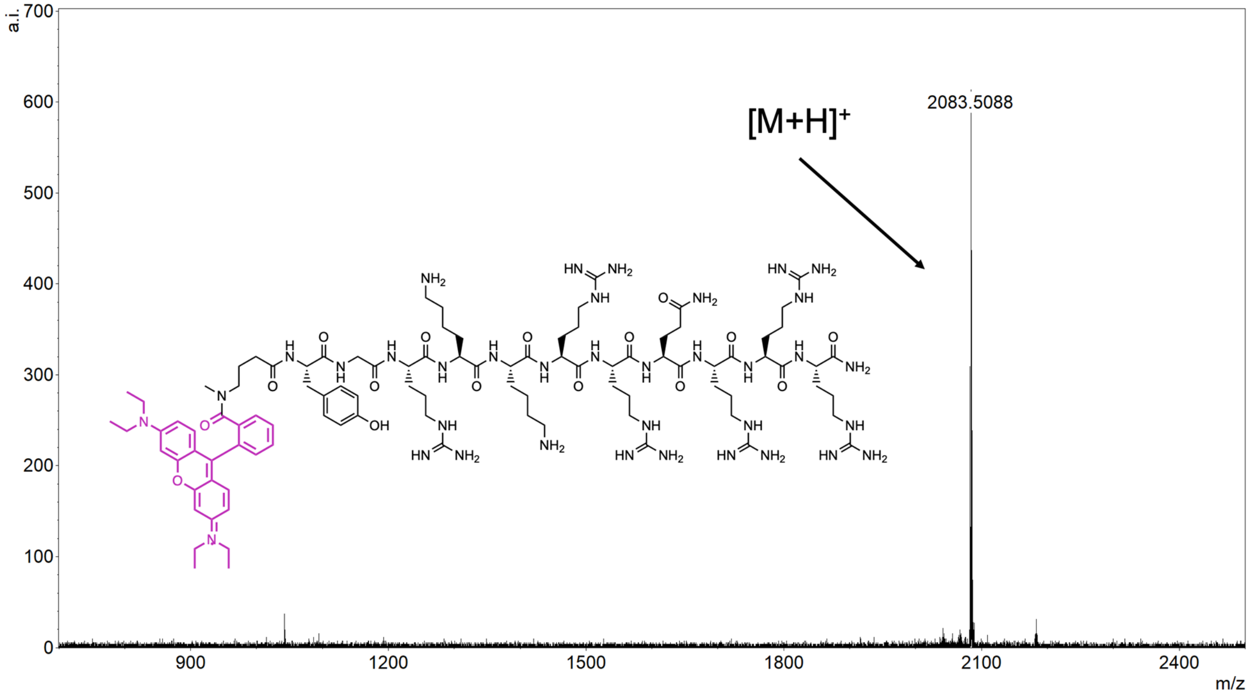


(**P**) Mass spectrum of RB-TAT47-57.

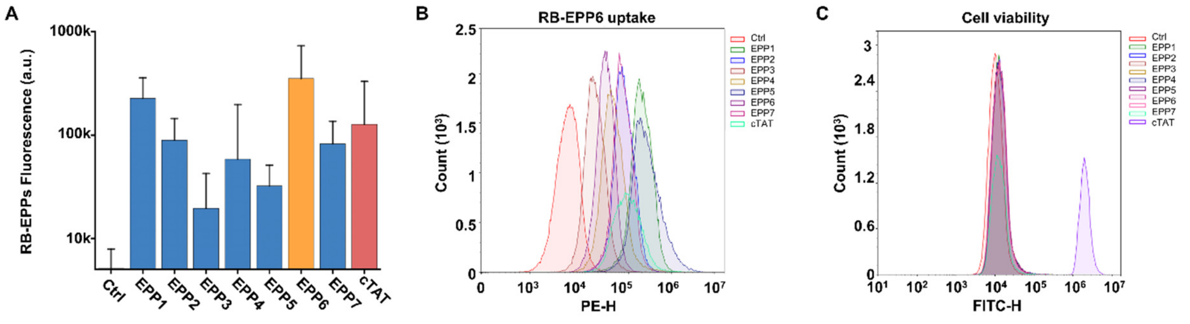


**Fig. S2.** Flow cytometry data demonstrate different fluorescence intensities. (**A**) Quantification of median RB fluorescence intensities. The Ctrl sample was untreated cells. The error bars denote standard deviation. (**B**) Distribution of the RB fluorescence (PE-H channel). (**C**) EPP incubation did not affect cell viability, as shown from the negative YOYO staining (FITC-H channel). cTAT appeared to affect cell viability (or membrane integrity) at this concentration.

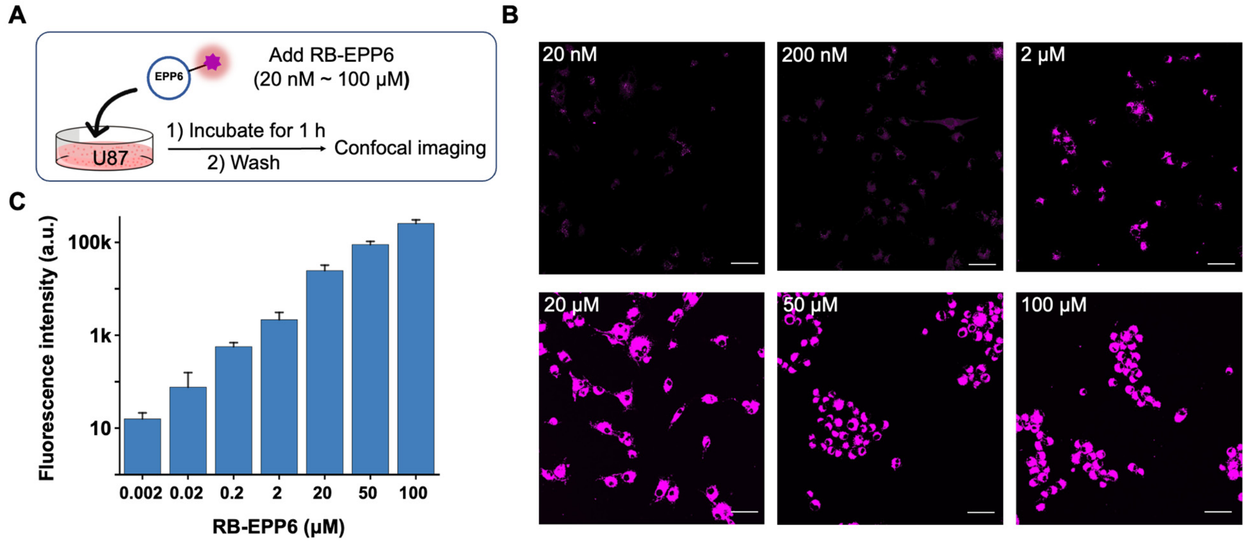


**Fig. S3.** RB-EPP6 enters U87 cells in a concentration-dependent manner. (**A**) Schematic illustration of the experimental procedure. (**B**) Confocal images showing the intracellular fluorescence signal in U87 cells. RB-EPP6 fluorescence is shown in magenta. The scale bars are 50 μm. (**C**) Fluorescence intensities extracted from the corresponding images. Around 30 representative cells were selected from each image, and the fluorescence intensities were extracted using Fiji (ImageJ) software. The error bars show the standard deviations.

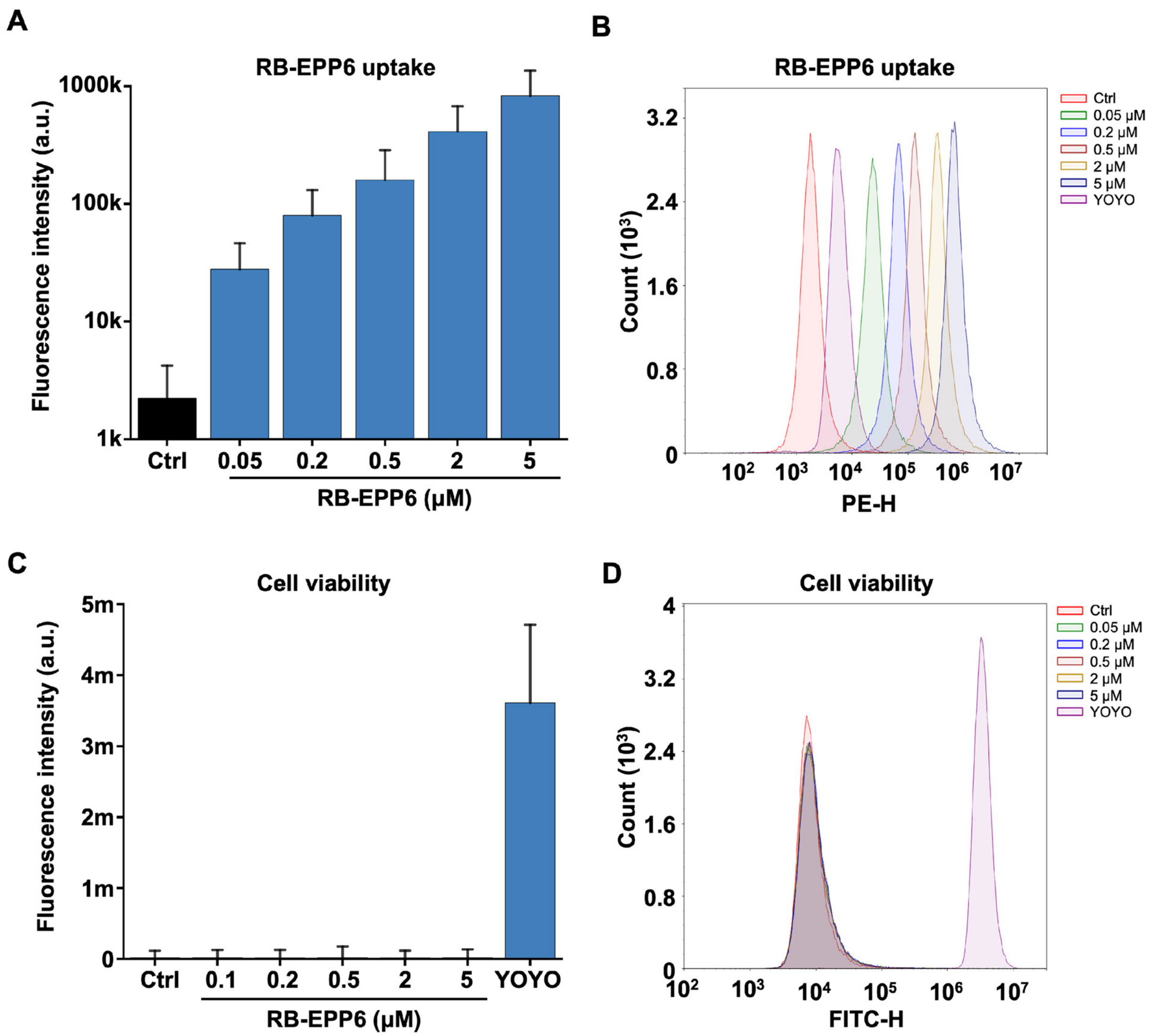


**Fig. S4.** Concentration dependence of RB-EPP6 uptake by U87 cells. (**A, B**) RB-EPP6 fluorescence intensities quantified by flow cytometry. The control sample (ctrl) was not treated with RB-EPP6. (**C, D**) RB-EPP6 did not affect cell viability. The YOYO sample shows the fluorescence intensities of fixed (dead) U87 cells. The error bars show the standard deviations of each measurement.

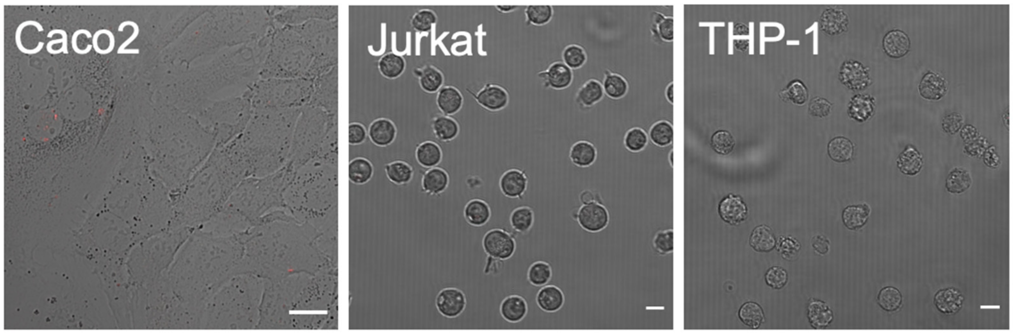


**Fig. S5.** Merged confocal images (bright field + RB channels) show no obvious RB-EPP6 uptake was observed in Caco2, Jurkat, and THP-1 cells. The scale bars are 10 µm.

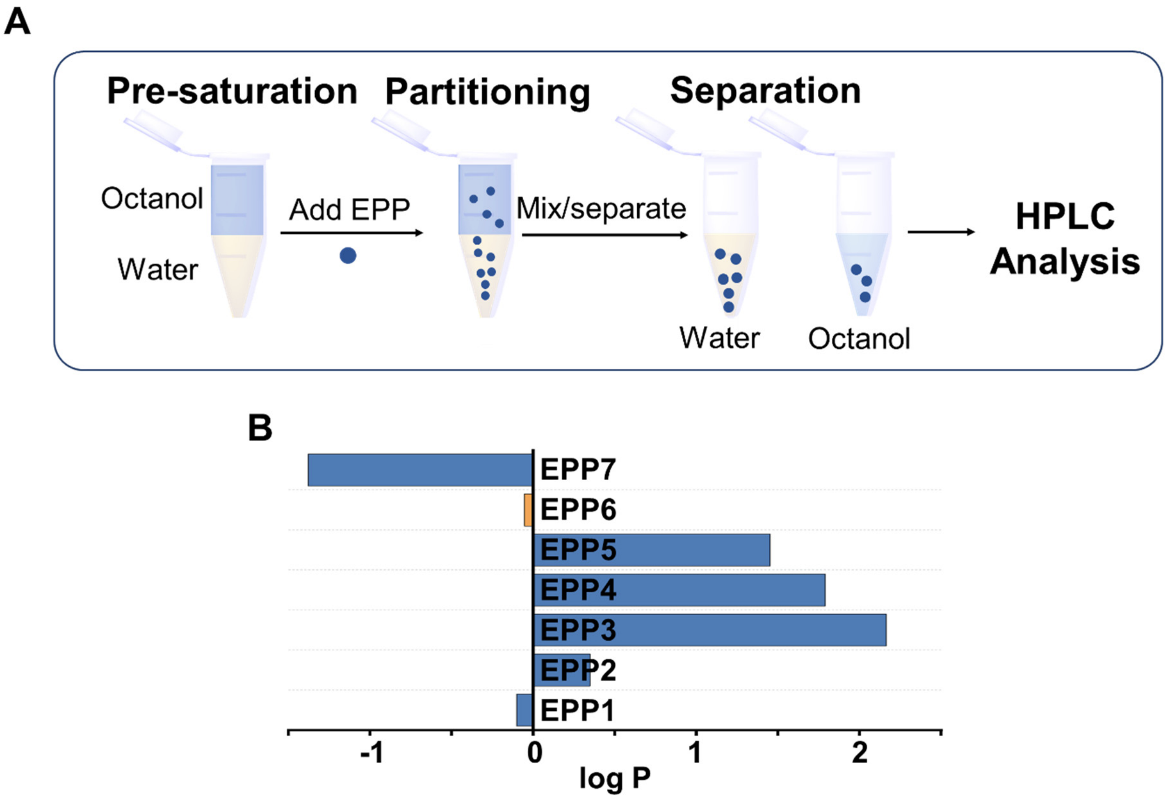


**Fig. S6.** (**A**) Schematic illustration of the octanol partitioning experiment. (**B**) Results of the octanol partitioning experiments showing that EPP6 was hydrophilic.

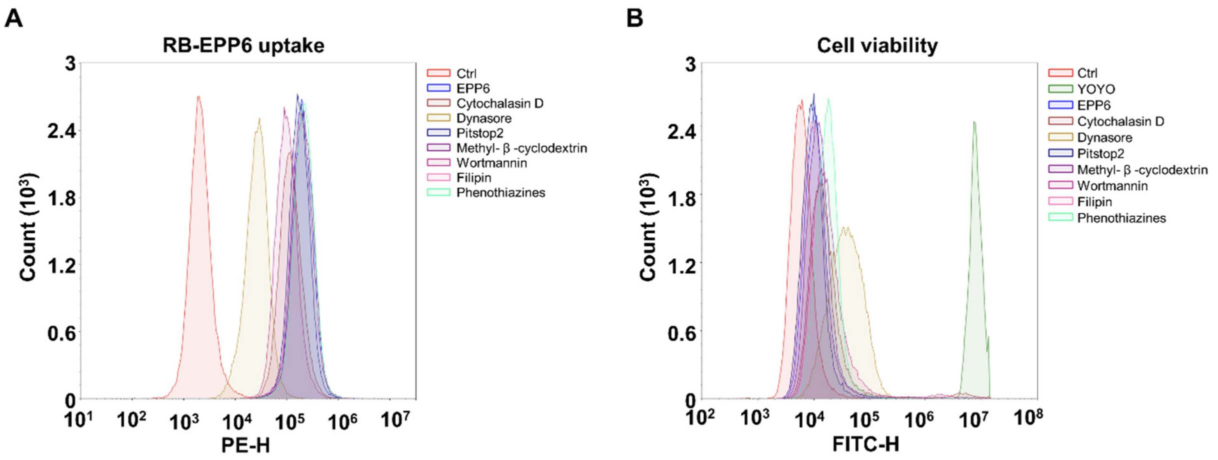


**C**

| Conditions | Median intensity (a.u.) | Percent change (%) |
|---|---|---|
| Ctrl | 2,508 | N/A |
| EPP6 | 222,200 | N/A |
| Cytochalasin D | 138,675 | -37.59 |
| Dynasore | 30,199 | -86.41 |
| Pitstop2 | 201,399 | -9.36 |
| Methyl-β-cyclodextrin | 234,850 | 5.69 |
| Wortmannin | 114,823 | -48.32 |
| Filipin | 220,201 | -0.90 |
| Phenothiazines | 242,075 | 8.94 |

**Fig. S7.** The effects of inhibitors on the RB-EPP6 uptake in U87 cells. (**A**) RB-EPP6 intensities (PE channel) quantified using flow cytometry. The control sample (ctrl) was not treated by RB-EPP6 and shows the background fluorescence intensity. (**B**) The corresponding cell viability quantified using the intracellular YOYO fluorescence (FITC channel). YOYO is a cell-impermeable dye that stains dead cells. The YOYO sample here shows the fluorescence intensity from fixed (dead) cells. (**C**) Median intensity and percent change (compared with EPP6) values extracted from the flow cytometry data.

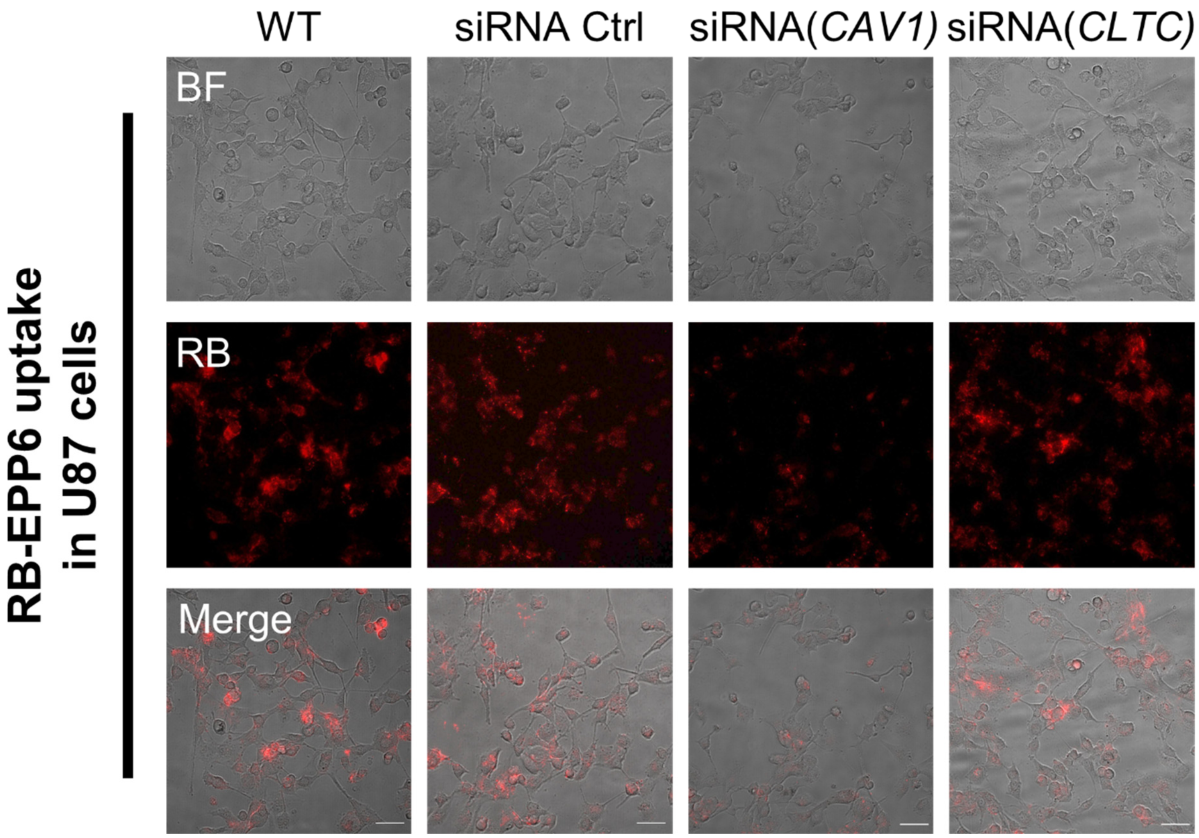


**Fig. S8.** Confocal images showing the intracellular fluorescence signal in siRNA-treated U87 cells. RB-EPP6 fluorescence is shown in red. The scale bars are 50 μm.

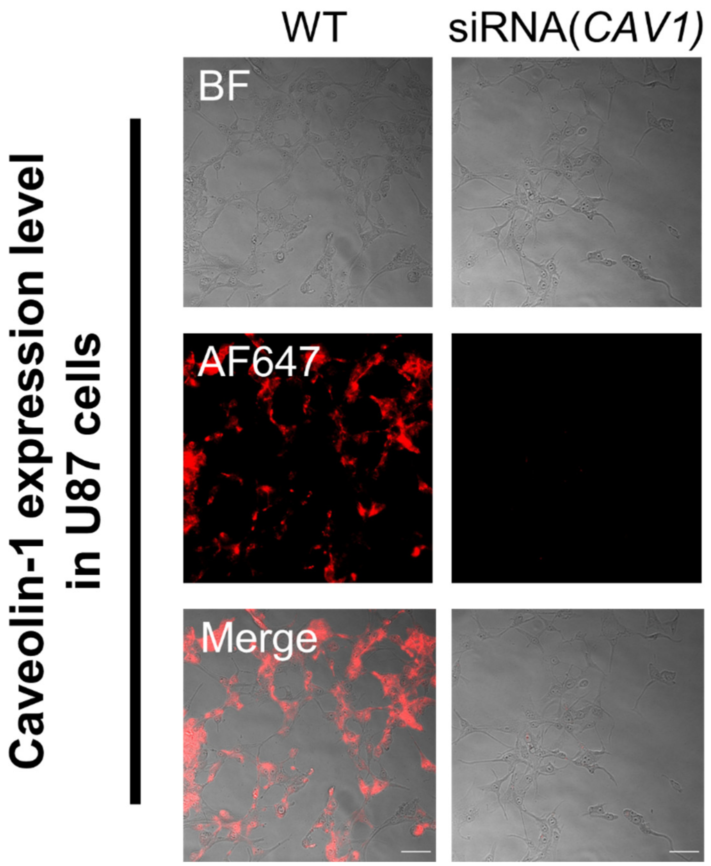


**Fig. S9.** Confocal images showing the caveolin-1 expression level in siRNA-treated U87 cells. AF647 fluorescence is shown in red. The scale bars are 50 μm.

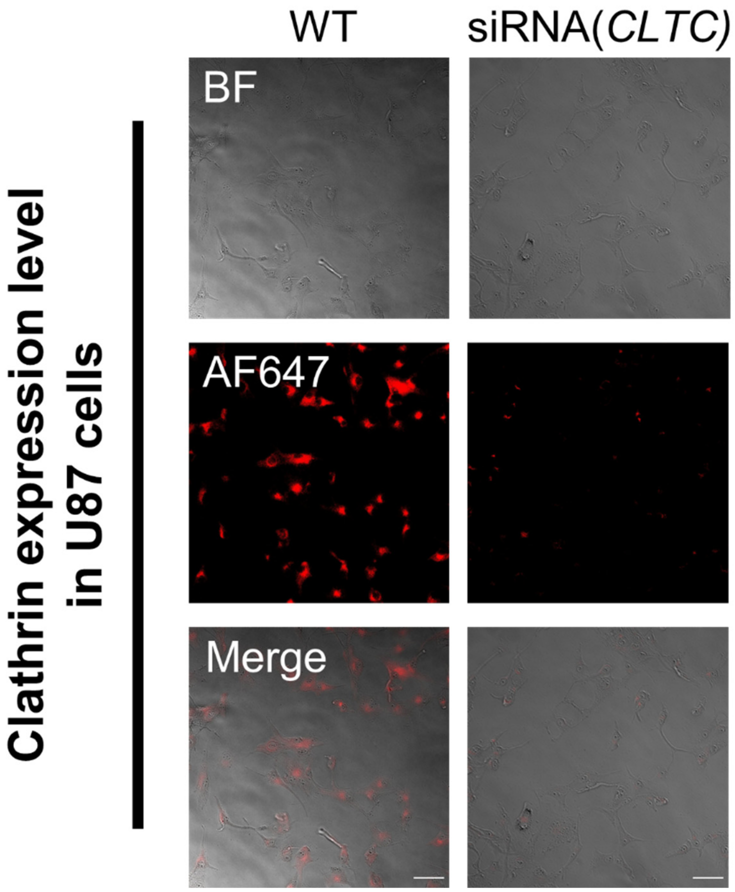


**Fig. S10.** Confocal images showing the clathrin expression level in *CLTC*-siRNA-treated U87 cells. AF647 fluorescence is shown in red. The scale bars are 50 μm.

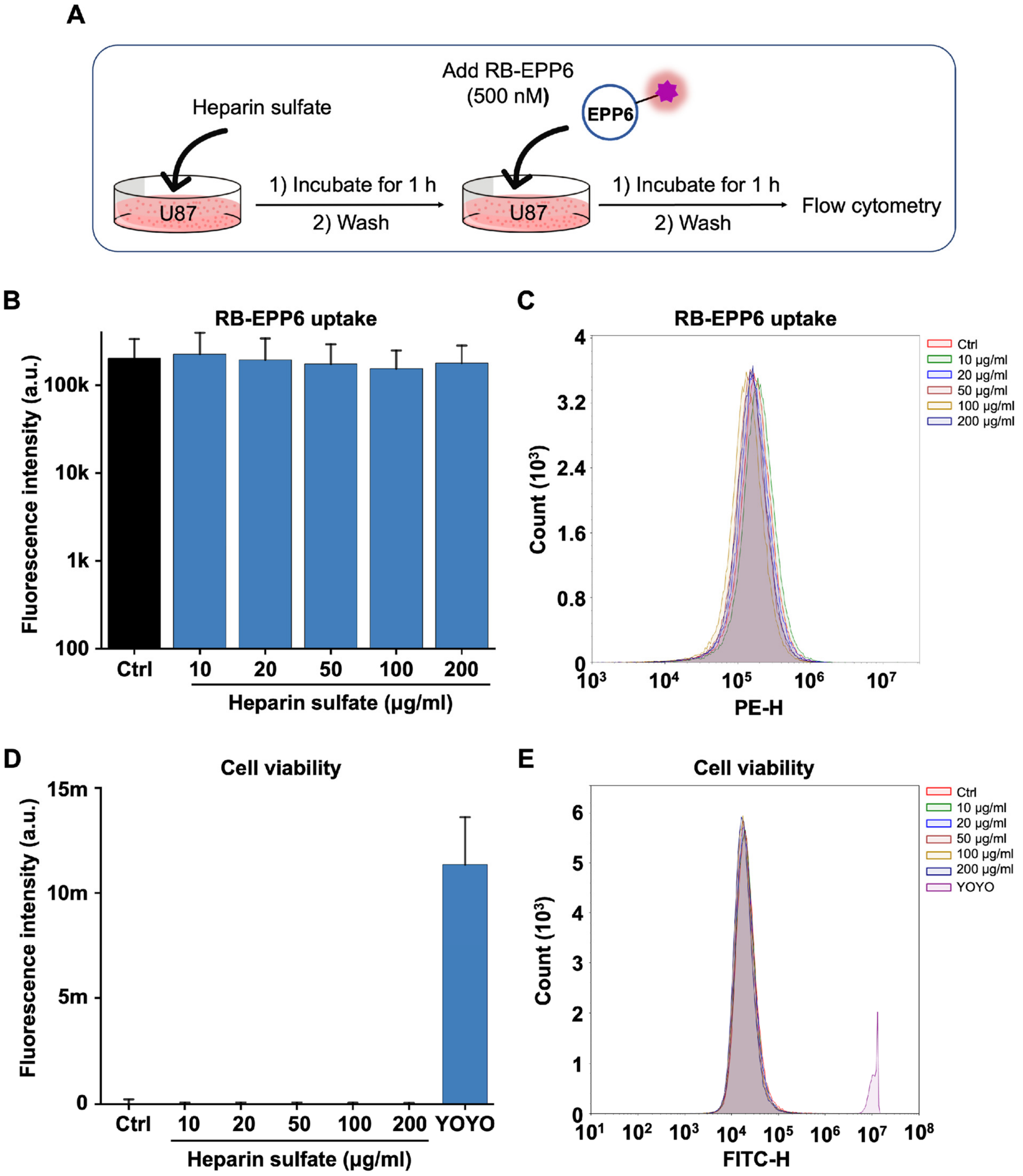


**Fig. S11.** Heparin sulfate treatment did not significantly affect RB-EPP6 uptake in U87 cells. (**A**) Schematic illustration of the experimental procedure. (**B, C**) RB-EPP6 fluorescence intensities quantified by flow cytometry. The control sample (ctrl) was treated with a buffer containing no heparin sulfate. (**D, E**) Heparin sulfate treatments did not affect cell viability. The YOYO sample shows the fluorescence intensities of fixed (dead) U87 cells. The error bars show the standard deviations of each measurement.

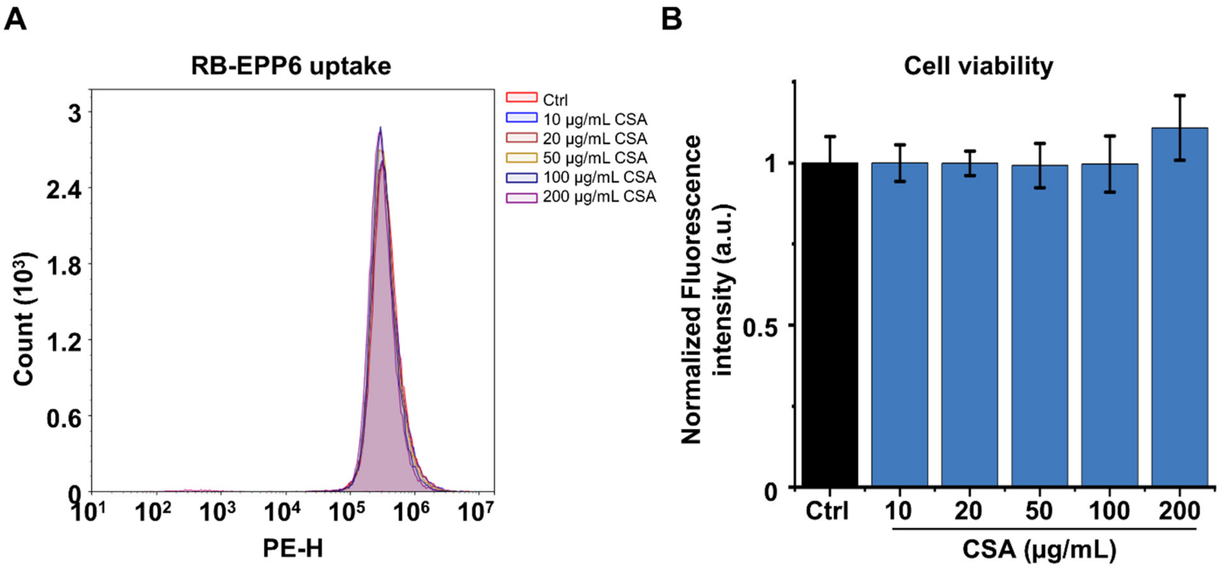


C

| CSA concentration (μg/mL) | Median intensity (a.u.) | Percent change (%) |
|---|---|---|
| Ctrl | 438,716 | 0 |
| 10 | 428,676 | -2.29 |
| 20 | 418,433 | -4.62 |
| 50 | 404,558 | -7.79 |
| 100 | 381,767 | -12.98 |
| 200 | 355,255 | -19.02 |

**Fig. S12.** (**A**) RB-EPP6 fluorescence intensities quantified by flow cytometry. The control sample (ctrl) was treated with a buffer containing no CSA. (**B**) Resazurin assay result shows that CSA treatments did not affect the viability of U87 cells. (**C**) Median intensity and percent change (compared with EPP6 Ctrl) values extracted from the flow cytometry data.

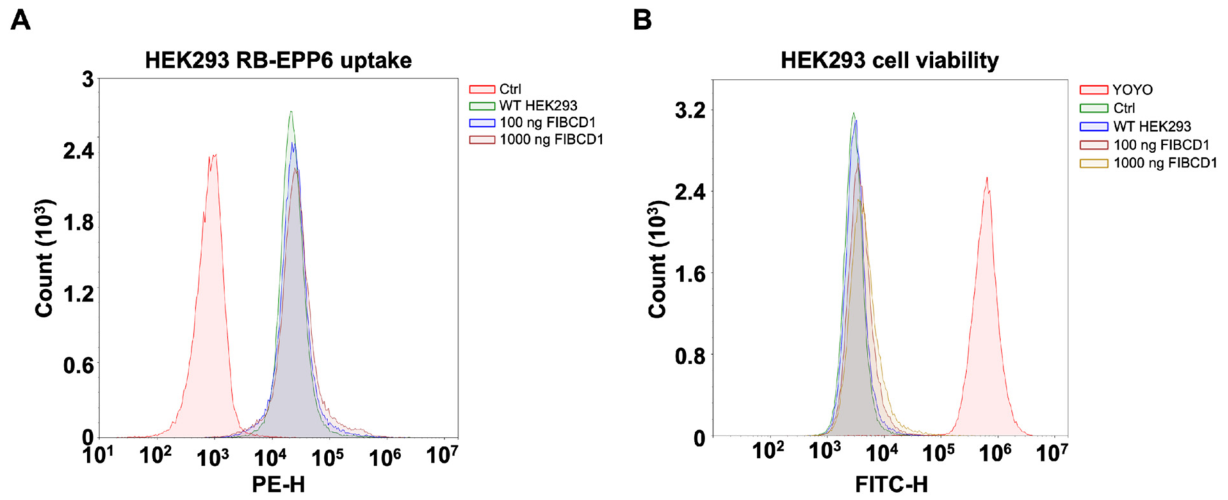


**Fig. S13.** FIBCD1 overexpression improves RB-EPP6 uptake in HEK293 cells. The cells were transfected with plasmids coding for full-length FIBCD1. (**A**) RB-EPP6 fluorescence intensity (PE channel) quantified by flow cytometry. The control sample (Ctrl) was not treated with RB-EPP6 and shows the fluorescence background. (**B**) FIBCD1 plasmid transfection did not affect cell viability, as demonstrated by the lack of YOYO signal (FITC channel). The YOYO sample shows the fluorescence intensity of fixed (dead) HEK293 cells.

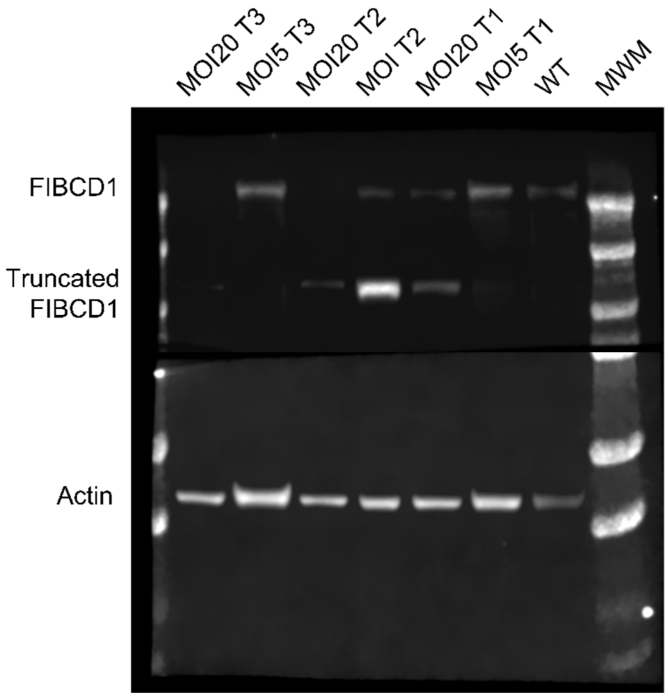


**Fig. S14.** Western blotting results showing the expression level of FIBCD1 and actin in stable cas9-U87 cells underwent CRISPR-based *FIBCD1*-KO selection. MWM, molecular weight markers. WT, wildtype control.

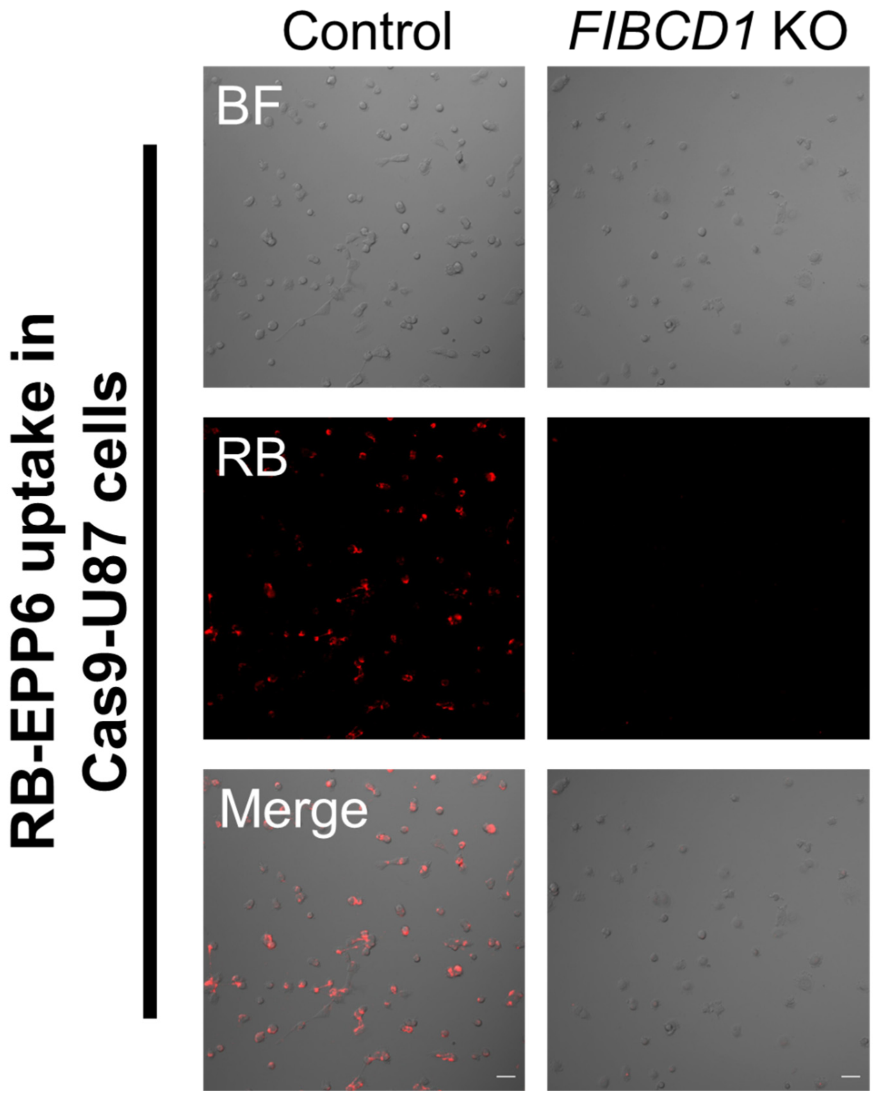


**Fig. S15.** Confocal images showing the RB-EPP6 uptake in cas9-U87 cells after CRISPR *FIBCD1*-KO. The scale bars are 50 μm.

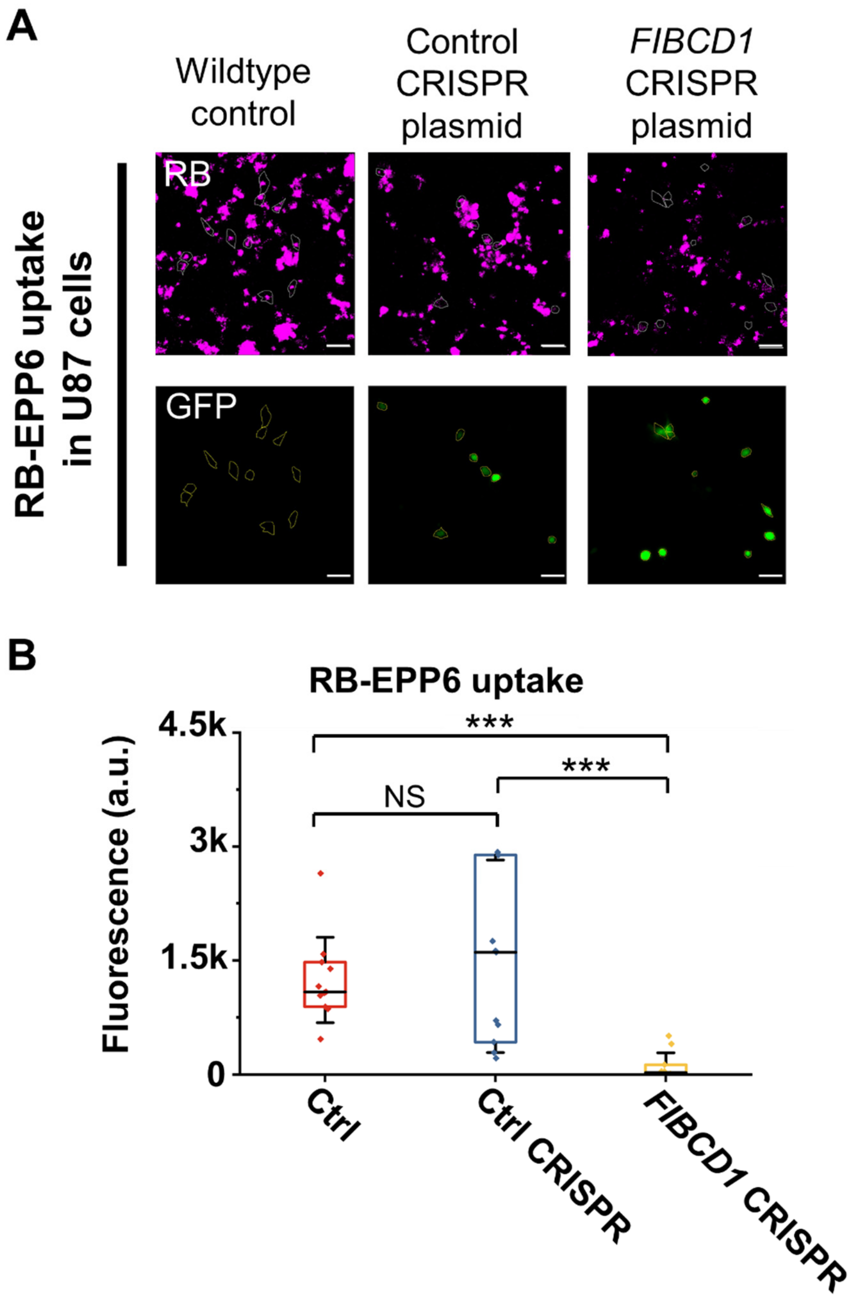


**Fig. S16.** Confocal imaging results showing that *FIBCD1*-KO by CRISPR obliterated RB-EPP6 uptake in U87 cells. (**A**) Representative confocal images. The control sample (Ctrl) was transfected with a CRISPR-Cas9 control plasmid that does not target the human proteome. Successful transfection was identified by the presence of GFP fluorescence. Yellow outlines show the representative cells chosen for analysis. The scale bars are 50 μm. (**B**) Quantification results. 11 representative single-cell data points were extracted from images for each sample. Mann-Whitney tests were used to evaluate the statistical significance. ***: $p < 0.001$. The boxes denote the middle two quartiles, the horizontal lines denote the median levels, and the whiskers show the standard deviations.

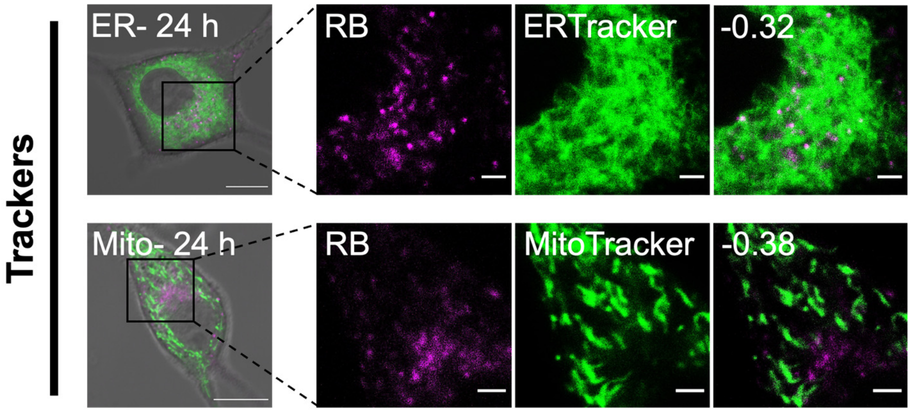


**Fig. S17.** RB-EPP6 did not enter the ER or the mitochondria. ERTracker and MitoTracker were used to label the organelles. The negative Pearson correlation values (right panels) show that the RB-EPP6 signal does not colocalize with the organelles. The scale bars show 10 µm in the original images on the left and 2 µm in the zoom-in images on the right.

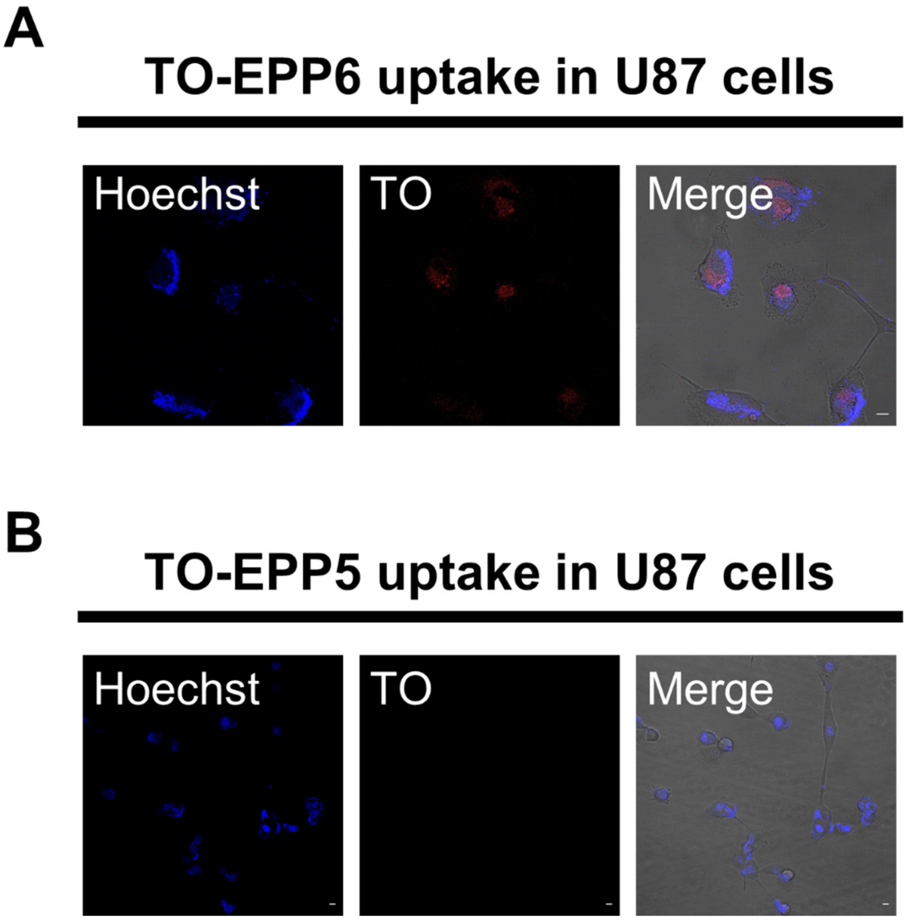


**Fig. S18.** (**A**) Thiazole orange-labeled EPP6 (TO-EPP6, 500 nM) showed nuclear accumulation in U87 cells, as demonstrated by the colocalization with the Hoechst signal. (**B**) Thiazole orange-labeled EPP5 (TO-EPP5, 500 nM) showed no uptake in U87 cells. Scale bar: 10 µm. These results prove that the TO moiety in TO-EPP6 was not the driving force for cell penetration or nuclear localization.

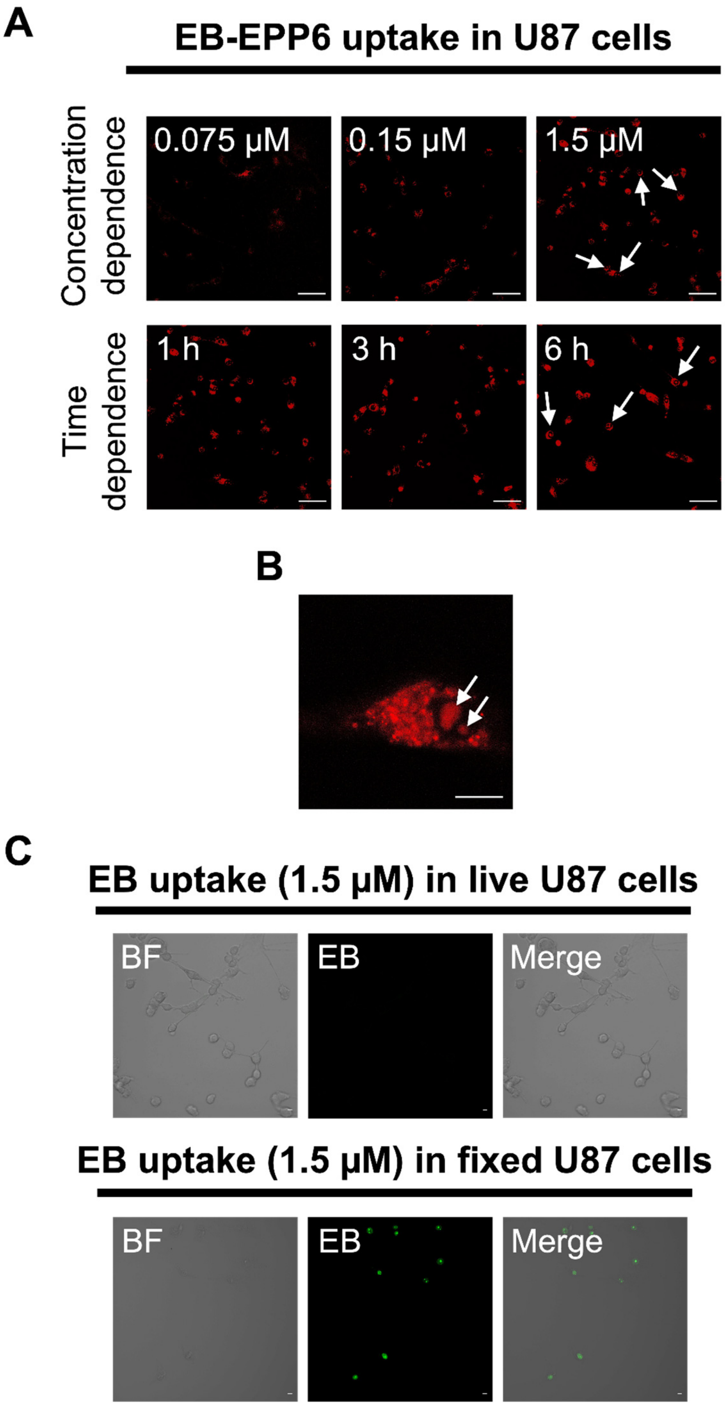


**Fig. S19.** Ethidium bromide-labeled EPP6 (EB-EPP6) showed nuclear accumulation. (**A**) Nuclear signals appeared in a concentration- and time-dependent manner. The concentration dependence experiment was conducted after 6 hr incubation of EB-EPP6. The time dependence experiment was conducted using 2 ug/mL EB-EPP6. The arrows point to representative cells with nuclear staining. (**B**) EB-EPP6 appeared in distinct nuclear compartments, possibly nucleoli, indicated by the arrows. (**C**) EB itself did not show any uptake in live U87 cells. On the other hand, EB signal was detected in fixed U87 cells. Scale bar: 10 µm.

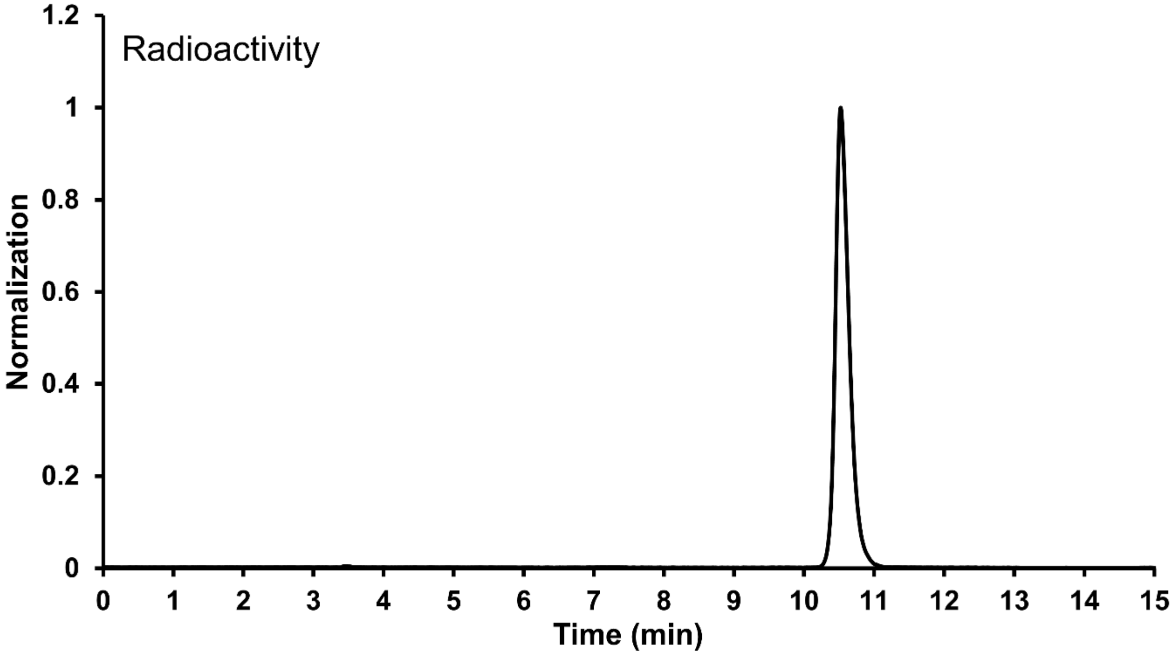

**Fig. S20**. Analytical radio-HPLC profile of $^{18}$F-**2**.

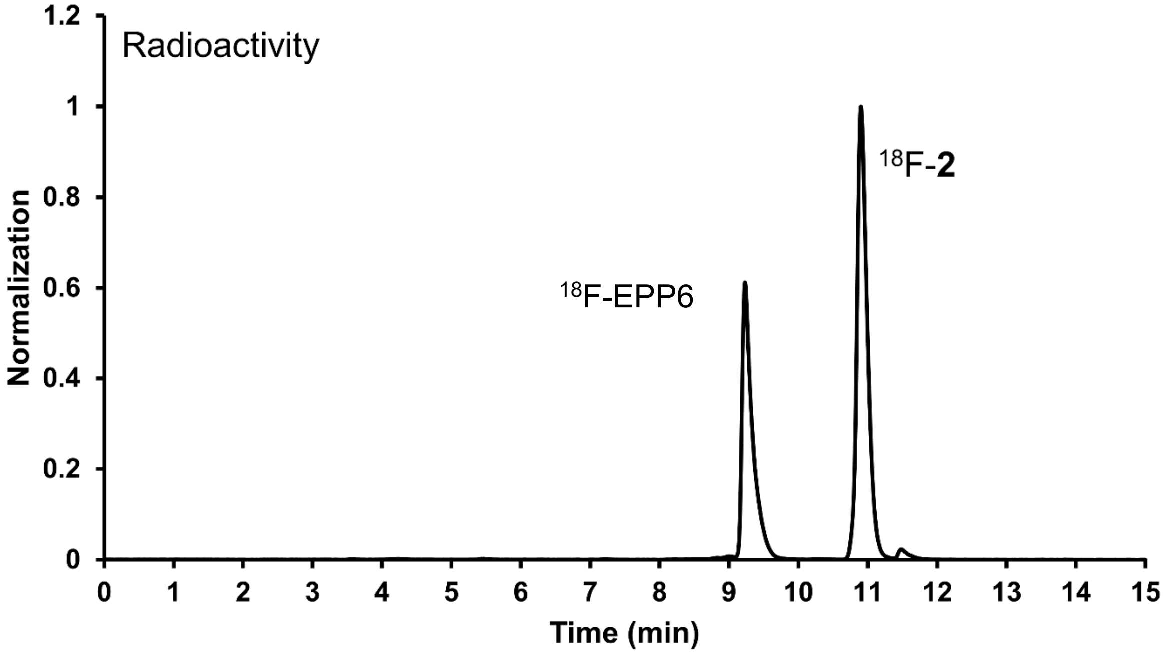


**Fig. S21.** Analytical radio-HPLC profile of crude mixture of $^{18}$F-EPP6 and $^{18}$F-**2**.

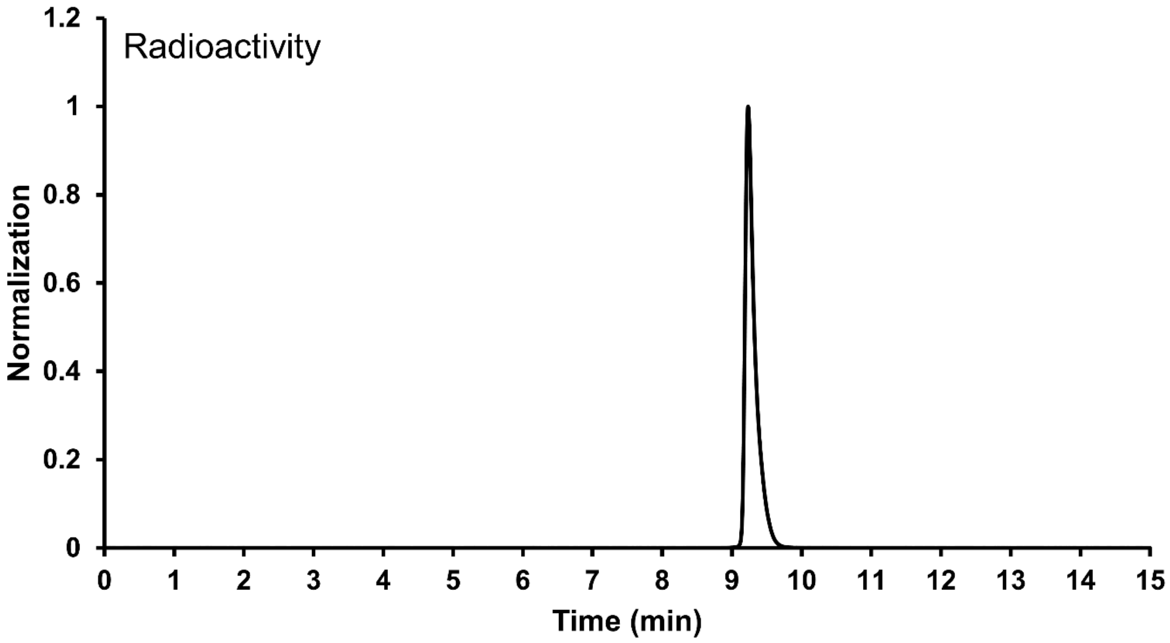


**Fig. S22.** Analytical radio-HPLC profile of $^{18}$F-EPP6.

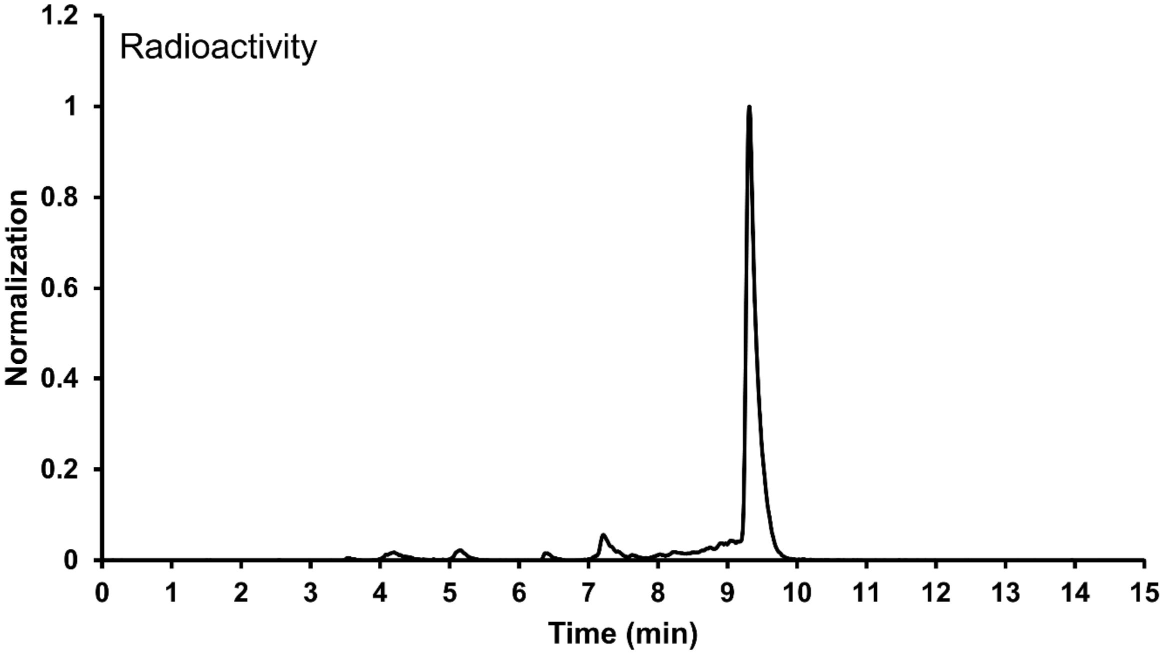


**Fig. S23**. Analytical radio-HPLC profile of $^{18}$F-EPP6 after incubation in mouse serum for 1 h.

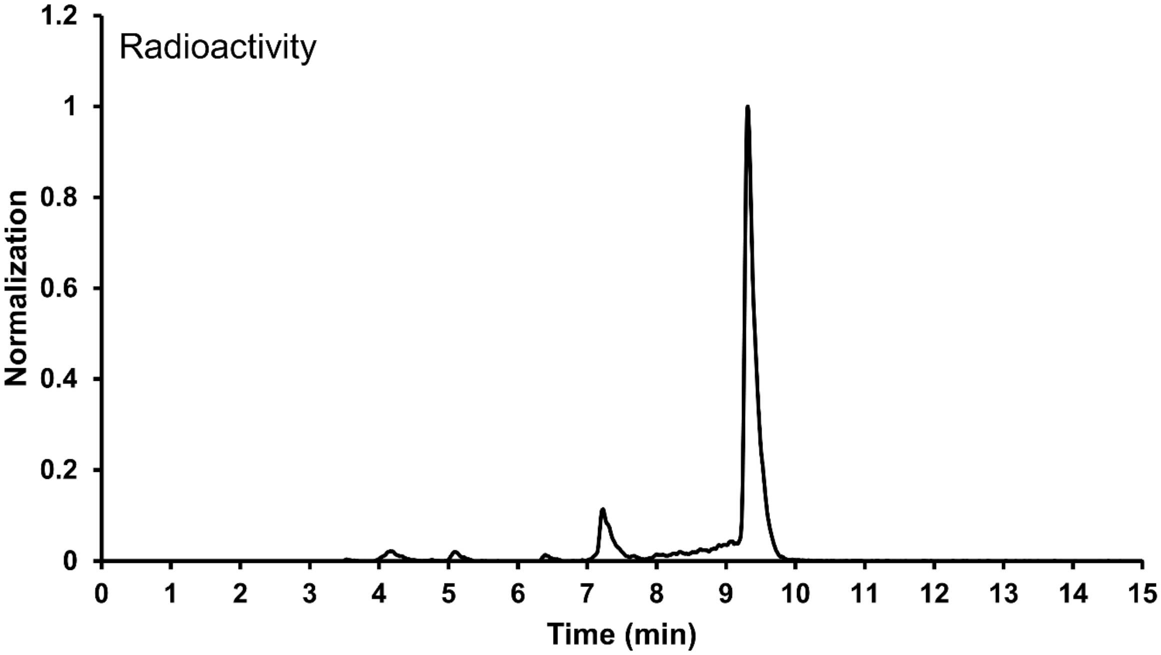

**Fig. S24**. Analytical radio-HPLC profile of $^{18}$F-EPP6 after incubation in mouse serum for 2 h.

**Table S1.** The calculated Pe and logPe values from the PAMPA assay.

| Compounds | Pe (cm/s) | logPe |
|---|---|---|
| **EPP3** | $1.12 \times 10^{-7}$ | -7.00 |
| **EPP1** | $1.62 \times 10^{-7}$ | -6.80 |
| **EPP6** | $2.34 \times 10^{-7}$ | -6.60 |
| **EPP2** | $4.69 \times 10^{-7}$ | -6.30 |
| **EPP7** | $6.32 \times 10^{-7}$ | -6.20 |
| **TAT** | $1.23 \times 10^{-6}$ | -5.90 |
| **EPP5** | $1.90 \times 10^{-6}$ | -5.70 |

**Table S2.** Up-regulated gene list comparing the high-uptake samples and the low-uptake samples.

| Gene name | log2 fold change | p-value (adjusted) | Role |
|---|---|---|---|
| ADAMTS4 | 1.43 | 3.83E-06 | Secreted |
| ATOH8 | 1.26 | 1.38E-22 | Intracellular |
| BDKRB2 | 1.02 | 2.25E-04 | GPCR |
| CA9 | 1.37 | 4.17E-19 | Rare protein |
| CAMK2A | 1.2 | 6.56E-04 | Intracellular |
| CEMIP | 1.17 | 1.39E-12 | Intracellular/Secreted |
| COL1A2 | 1.37 | 9.68E-34 | Collagen |
| COL5A1 | 1.15 | 3.25E-13 | Collagen |
| CYP26B1 | 2.51 | 4.51E-18 | Intracellular |
| DCHS2 | 1.08 | 7.79E-04 | Rare protein |
| DLX5 | 1.94 | 7.74E-10 | Intracellular |
| EGR2 | 1.04 | 2.57E-12 | Intracellular |
| ELFN2 | 1.38 | 1.31E-04 | Intracellular |
| FAT2 | 1.59 | 1.82E-09 | Cadherin |
| FENDRR | 1.03 | 1.32E-13 | Noncoding RNA |
| FER1L4 | 1.38 | 6.64E-08 | Noncoding RNA |
| **FIBCD1** | 1.33 | 9.26E-05 | Surface receptor |
| FN1 | 1.82 | 3.63E-74 | Intracellular/Secreted |
| GALNT5 | 1.93 | 2.39E-14 | Intracellular |
| HMCN1 | 1.46 | 2.00E-22 | Secreted |
| HSPB6 | 1.02 | 8.30E-11 | Intracellular |
| LAMA4 | 1.11 | 1.01E-19 | Laminin |
| LOC101448202 | 1.19 | 3.79E-10 | RNA Gene |
| LRRC15 | 1.34 | 6.22E-36 | Rare protein |
| MFAP4 | 1.02 | 3.89E-13 | Secreted |
| NOS1 | 1.74 | 9.61E-09 | Nitric Oxide Synthase 1 |
| PPP2R2B | 1.17 | 6.12E-06 | Intracellular |
| RARB | 1.02 | 1.76E-06 | Intracellular |
| ROS1 | 1.51 | 6.68E-04 | Intracellular |
| RTBDN | 1.16 | 6.83E-05 | Secreted |
| SLC26A4 | 1.37 | 1.66E-06 | Solute carrier |
| SSC5D | 1.05 | 8.30E-11 | Intracellular/Secreted |
| SYNDIG1 | 1.19 | 1.90E-08 | Intracellular |
| TENM2 | 1.06 | 4.27E-07 | Intracellular |
| TMEM119 | 1.63 | 4.45E-16 | Rare protein |
| TSPAN9 | 1.01 | 2.91E-10 | Nucleoplasm |
| WDR63 | 1.08 | 8.13E-05 | Intracellular |
| WISP1 | 1.76 | 1.04E-07 | Intracellular/Secreted |
